# Supplementary material for: Dimeric G-quadruplex motifs-induced NFRs determine strong replication origins in vertebrates
Source: Nat Commun. 2023 Aug 10;14:4843. doi: 10.1038/s41467-023-40441-4 (PMC10415359; doi:10.1038/s41467-023-40441-4)

a

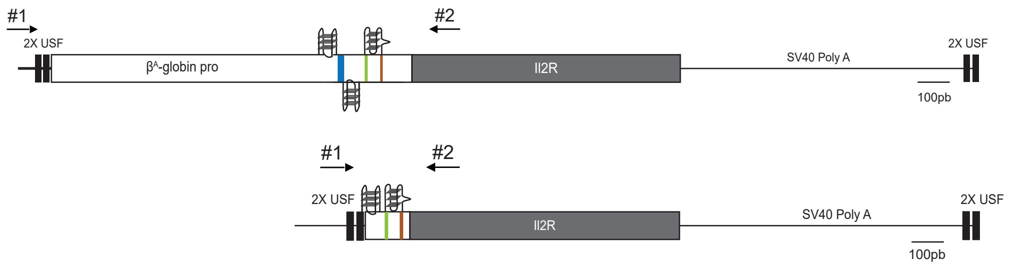

b

Relative quantification values		*Med14* gene	*IL2R* gene
RT+-qPCR	βA-globin full origin (1)	100	0.04
	βA-globin full origin (2)	100	0.03
	βA-globin minimal origin (1)	100	0.04
	βA-globin minimal origin (2)	100	0.01

Crossing point values		*Med14 gene*	*IL2R gene*
RT+-qPCR	βA-globin full origin (1)	22.28	35.41
	βA-globin full origin (2)	21.38	35.14
RT--qPCR	βA-globin full origin (1)	NA	NA
	βA-globin full origin (2)	NA	NA
RT+-qPCR	βA-globin minimal origin (1)	22.03	35.27
	βA-globin minimal origin (2)	21.03	36.46
RT--qPCR	βA-globin minimal origin (1)	NA	NS
	βA-globin minimal origin (2)	NA	NA

c

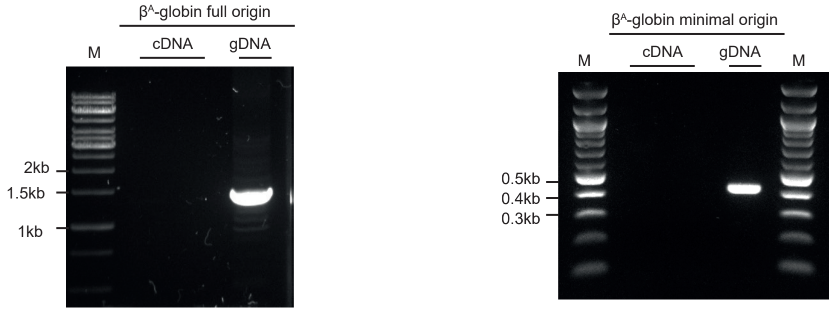

Supplementary Figure 1: The full and minimal βA-globin origins do not drive transcription
(a) Schemes of the full and minimal βA-globin origins coupled with the IL2R gene, SV40 PolyA sequence and USF binding sites are shown. Positions of specific primers (#1 and #2) used to detect potential RNAs elongated over pG4s #1 and #3 are indicated with arrows. (b) Relative quantification by RT-qPCR on mRNAs was performed in clonal cell lines containing the full and minimal βA-globin origins. mRNA levels were normalized against Med14 mRNA levels arbitrarily set at 100 (first table). Crossing point values for RT+-qPCR and RT--qPCR (background levels) experiments are reported in the second table. NA and NS correspond to non-amplified and non-specific signals respectively. Source data are provided as a Source Data file. (c) PCR products obtained with primers #1 and #2 to amplify either cDNA (cDNA) or genomic DNA (gDNA) as a control were subjected to electrophoresis in a 1% w/v agarose gel and stained with SYBR safe. The DNA size marker used is a commercial 1 kb plus DNA ladder (M, left panel) or a 100 bp DNA ladder (M, right panel). The absence of the 1.3 kb or the 0.4 kb PCR products after amplification over pG4 #1 and #3 with primers #1 and #2 in cDNAs from the full and minimal βA-globin origins demonstrates the absence of transcription at these sites.

a

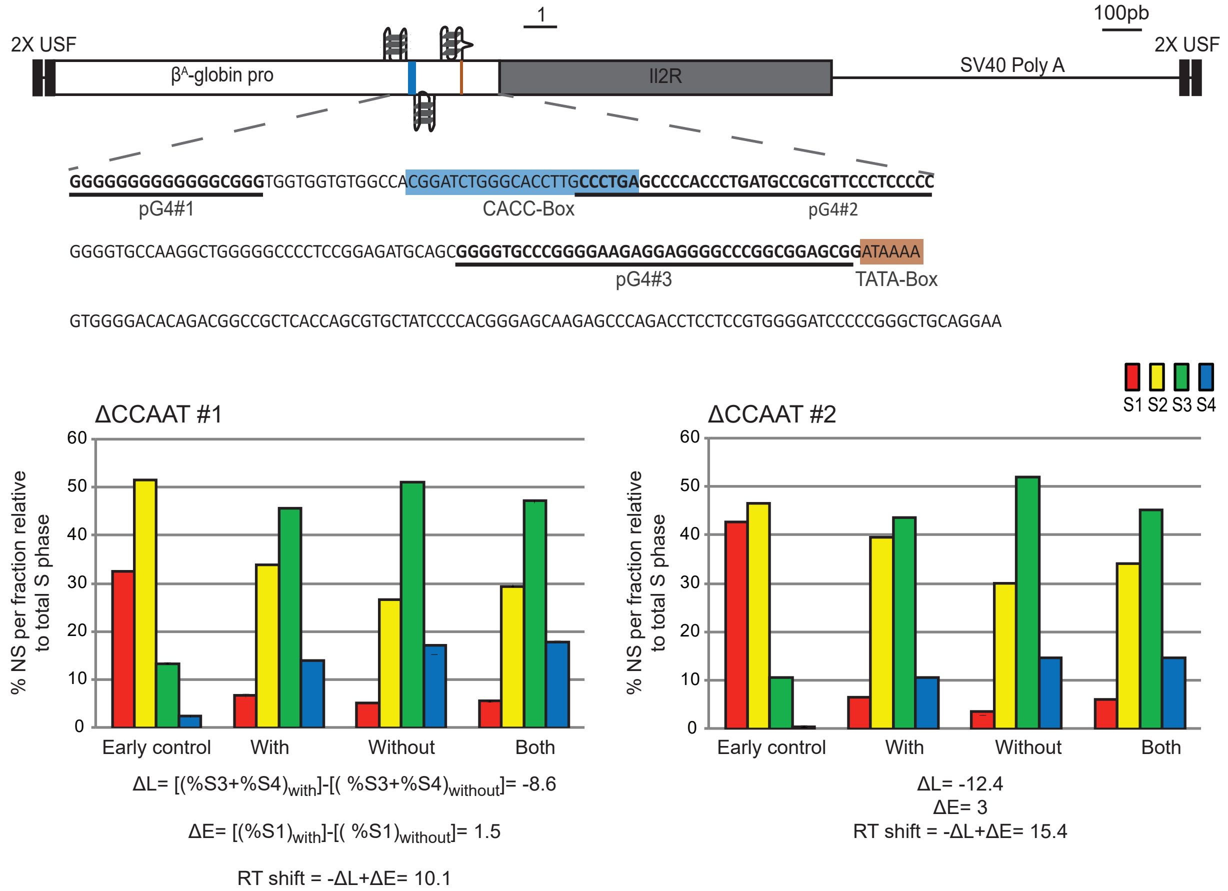

b

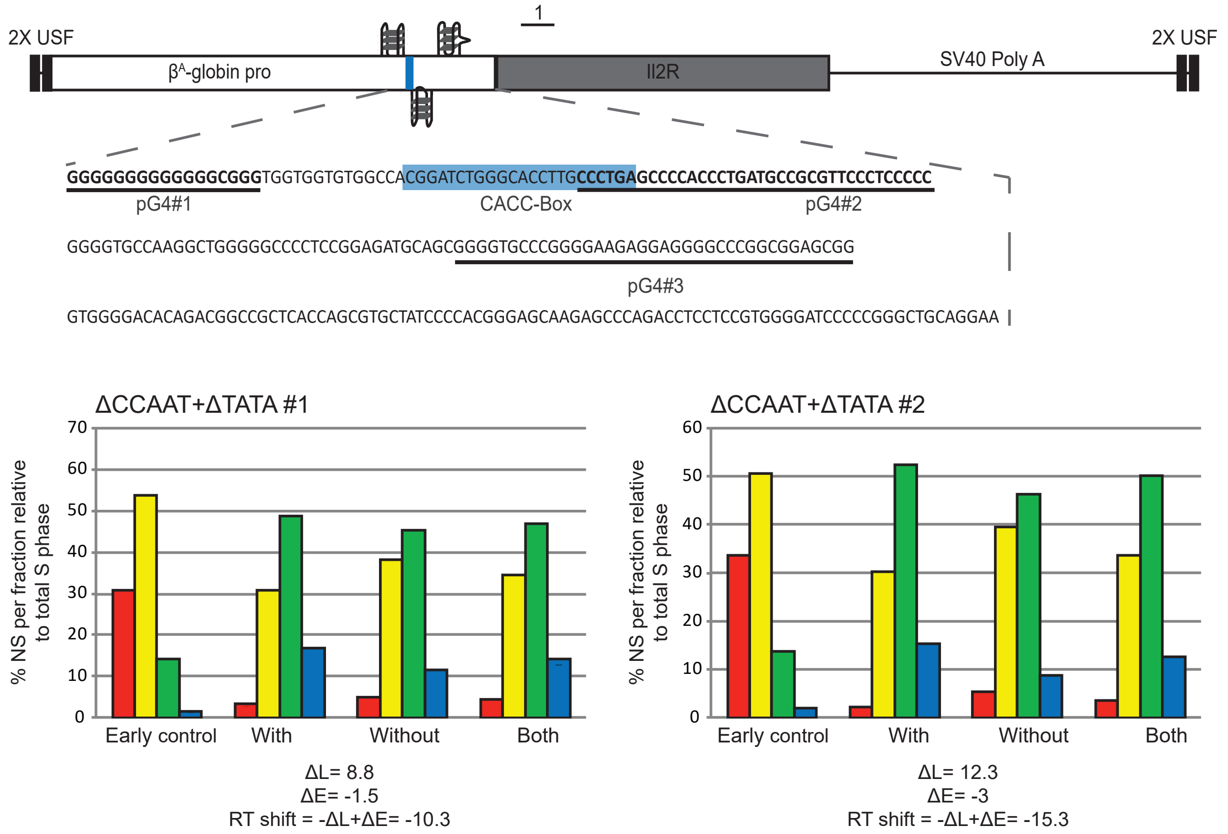

Supplementary Figure 2: RT analyses of the ΔCCAAT and ΔCCAAT+ΔTATA β^{A}-globin origins
(a-b) Schemes of the ΔCCAAT β^{A}-globin origin (a) and the ΔCCAAT+ΔTATA β^{A}-globin origin (b) coupled with the IL2R gene, SV40 PolyA sequence and USF binding sites are shown on the top. The corresponding sequences are given below each scheme with pG4s underlined and cis-regulatory elements delineated by colored boxes (blue for the CACC-Box and brown for the TATA-Box). Analyses of two ΔCCAAT β^{A}-globin origin clonal cell lines (a) or two ΔCCAAT+ΔTATA β^{A}-globin origin clonal cell lines (b) are reported. RT profiles of each chromosomal allele are determined after targeted transgene integration using the allele-specific analysis of RT method by real-time PCR quantification (1). BrdU pulse-labeled cells were sorted into four S-phase fractions from early to late (S1 to S4) and the immune-precipitated newly synthesized strands (NS) were quantified by real-time qPCR in each fraction. Specific primer pairs determine the RT profile for the modified allele (With, black line, 1), the wt allele (Without) and both alleles (Both). The endogenous β-globin locus was analyzed as an early-replicated control (Early). Differences in -ΔL+ΔE values calculated at the target site following transgene integration are indicated. Error bars correspond to the standard deviation for qPCR duplicates. Source data are provided as a Source Data file.

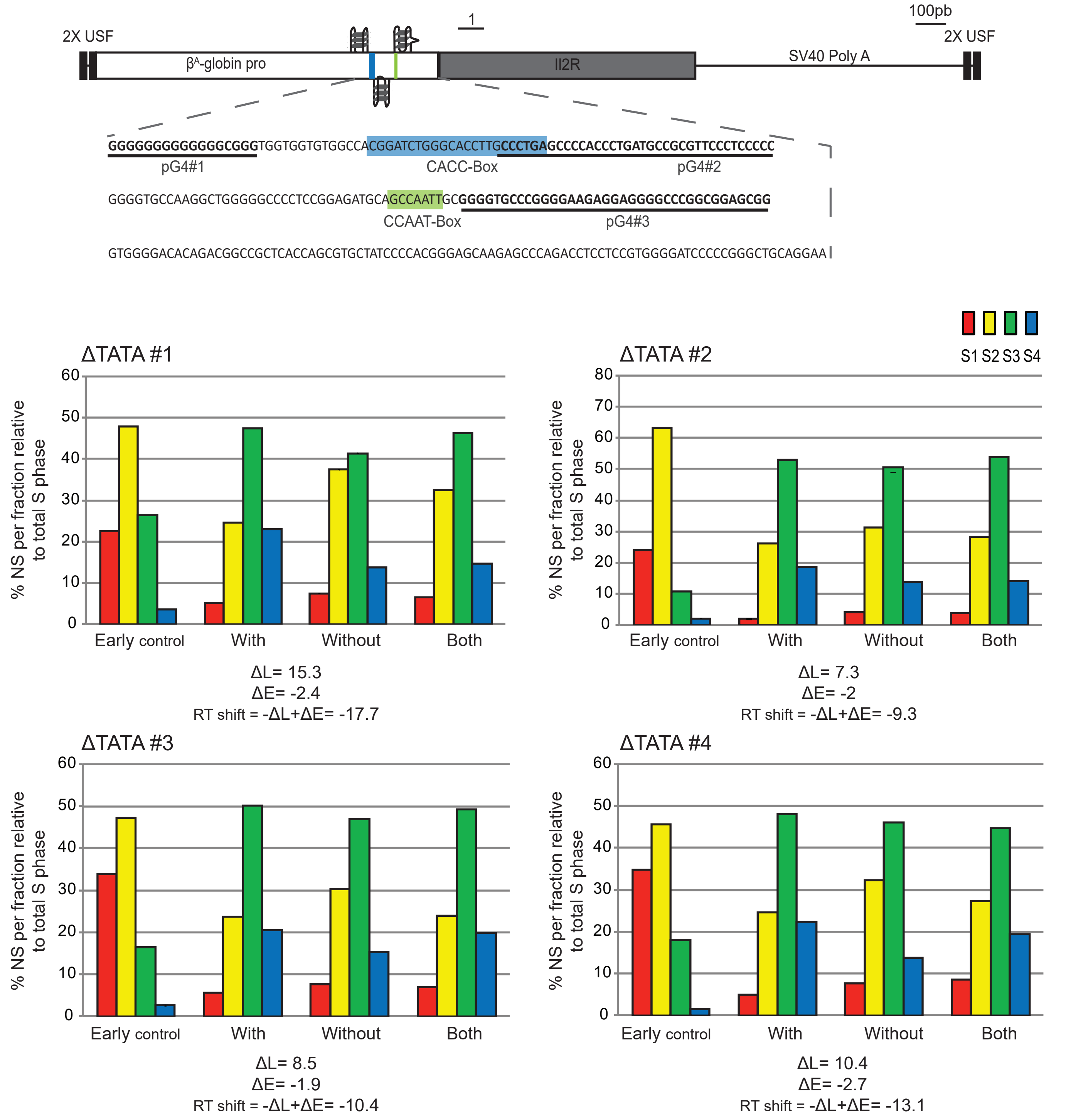

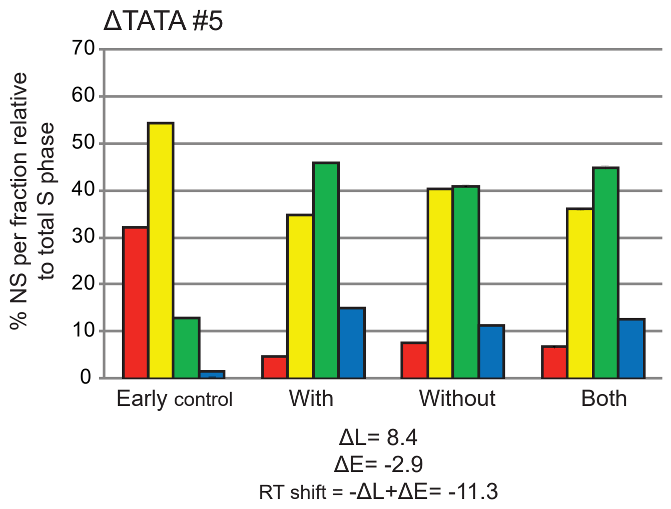

Supplementary Figure 3: RT analyses of the ΔTATA β^A-globin origin
Scheme of the β^A-globin ΔTATA origin coupled with the IL2R gene, SV40 PolyA sequence and USF binding sites is shown on the top. The corresponding sequence is given below with pG4s underlined and *cis*-regulatory elements delineated by colored boxes (blue for the CACC-Box and green for the CCAAT-Box). Analyses of five ΔTATA β^A-globin origin clonal cell lines are reported. RT profiles of each chromosomal allele is determined after targeted transgene integration using the allele-specific analysis of RT method by real-time PCR quantification as described in supplementary figure 2. Source data are provided as a Source Data file.

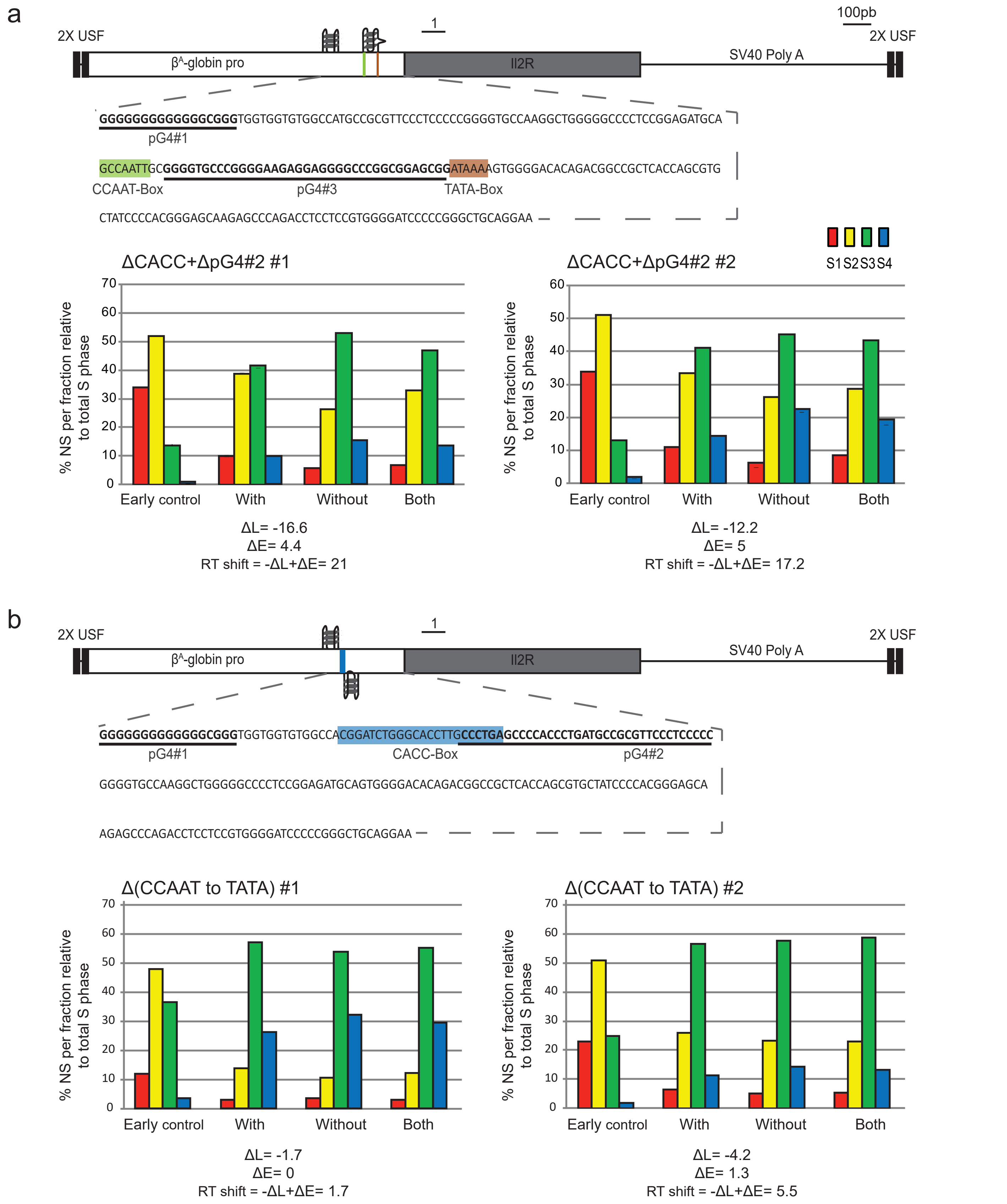

Supplementary Figure 4: RT analyses of the ΔCACC+pG4#2 and Δ(CCAAT to TATA) β^A-globin origins
(a-b) Schemes of the β^A-globin ΔCACC+pG4#2 origin (a) and the β^A-globin Δ(CCAAT to TATA) origin (b) coupled with the IL2R gene, SV40 PolyA sequence and USF binding sites are shown on the top. The corresponding sequences are given below each scheme with pG4s underlined and cis-regulatory elements delineated by colored boxes (blue for the CACC-Box, green for the CCAAT-Box and brown for the TATA-Box). Analyses of two ΔCAACC+pG4#2 β^A-globin origin clonal cell lines (a) or two Δ(CCAAT to TATA) β^A-globin origin clonal cell lines (b) are reported. RT profiles of each chromosomal allele are determined after targeted transgene integration using the allele-specific analysis of RT method by real-time PCR quantification as described in supplementary figure 2. Source data are provided as a Source Data file.

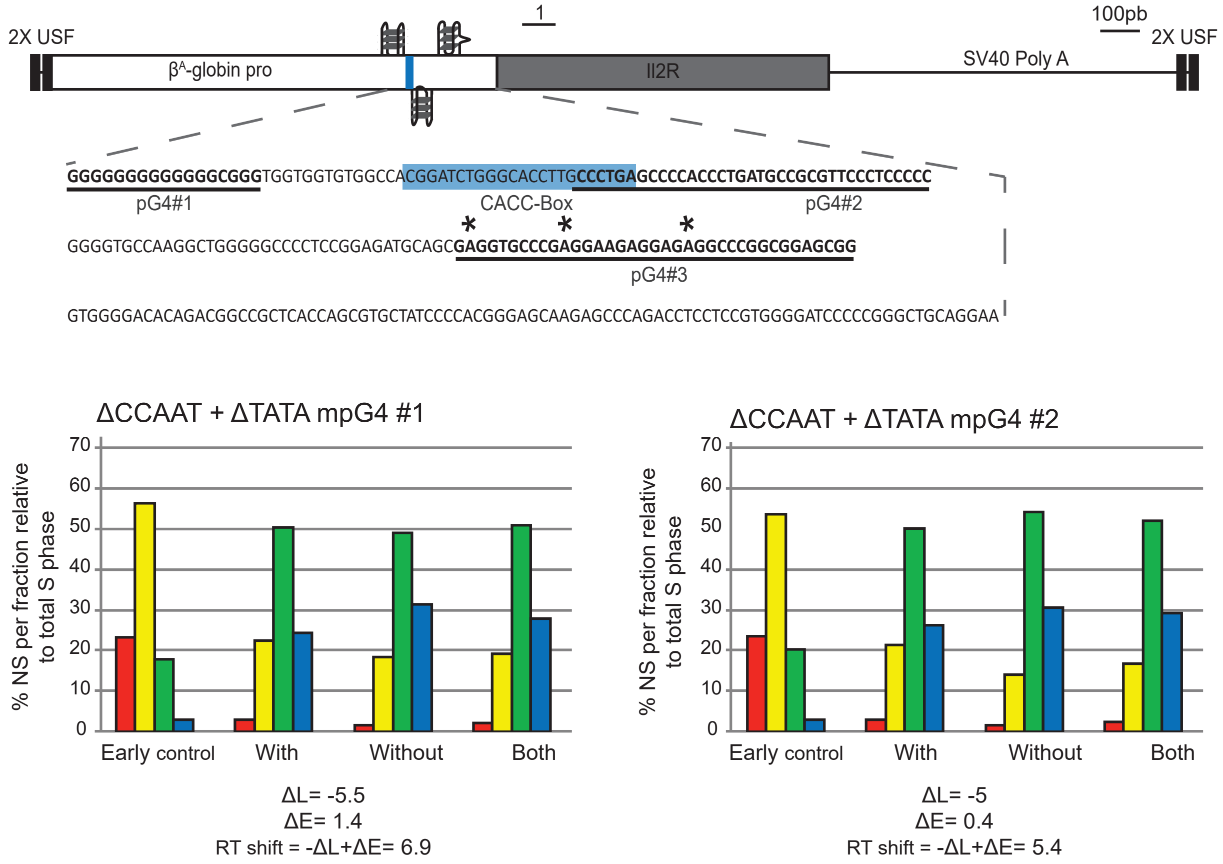

Supplementary Figure 5: RT analyses of the ΔCCAAT+ΔTATA mpG4 β^{A}-globin origin

Scheme of the β^{A}-globin ΔCCAAT+ΔTATA mpG4 origin coupled with the IL2R gene, SV40 PolyA sequence and USF binding sites is shown at the top. The corresponding sequence is given below with pG4s underlined and cis-regulatory elements delineated by colored boxes (blue for the CACC-Box). Points mutation inside the pG4#3 are indicated with black stars. Analyses of two ΔCCAAT+ΔTATA β^{A}-globin origin clonal cell lines are reported. RT profiles of each chromosomal allele is determined after targeted transgene integration using the allele-specific analysis of RT method by real-time PCR quantification as described in supplementary figure 2. Source data are provided as a Source Data file.

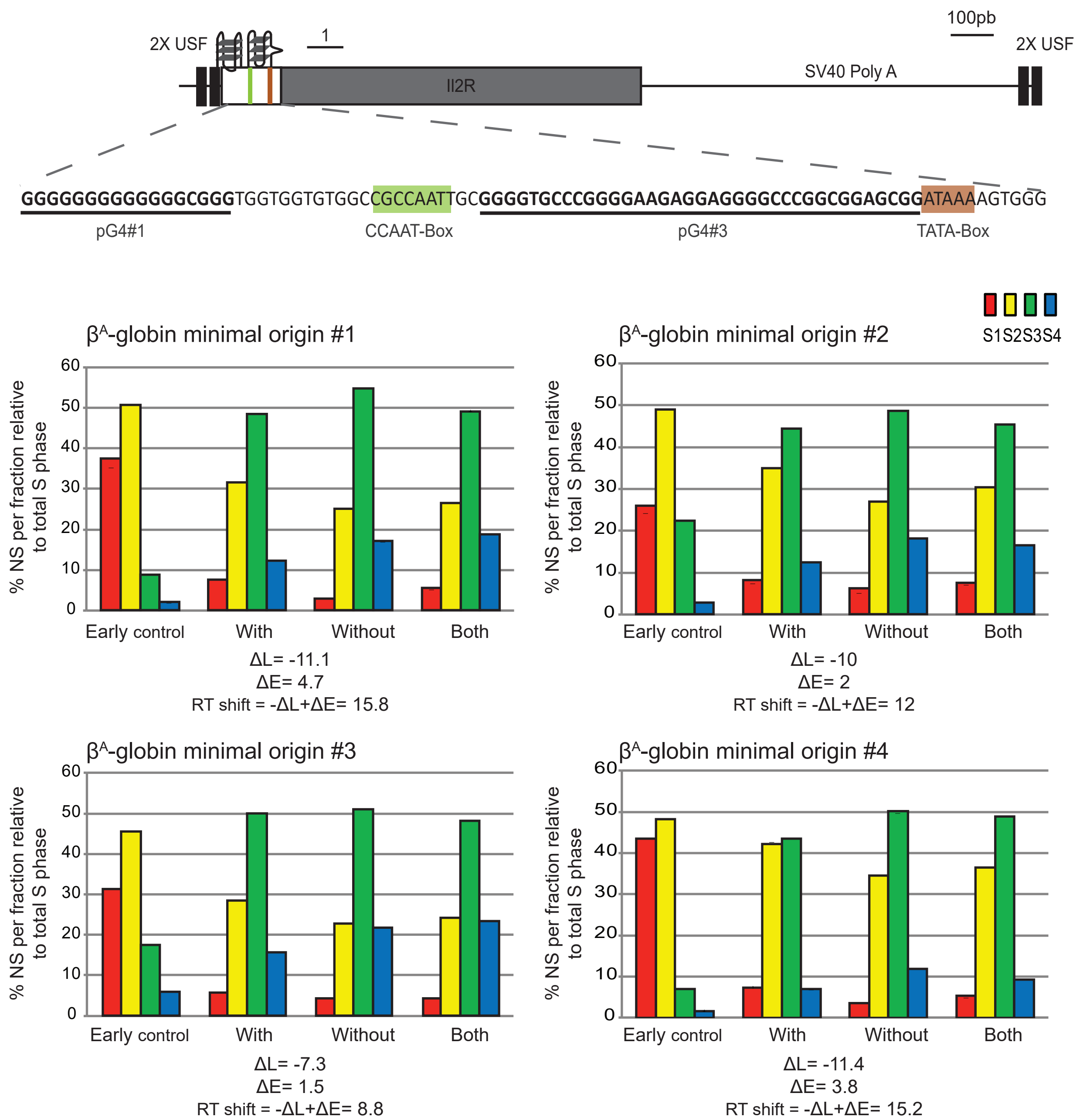

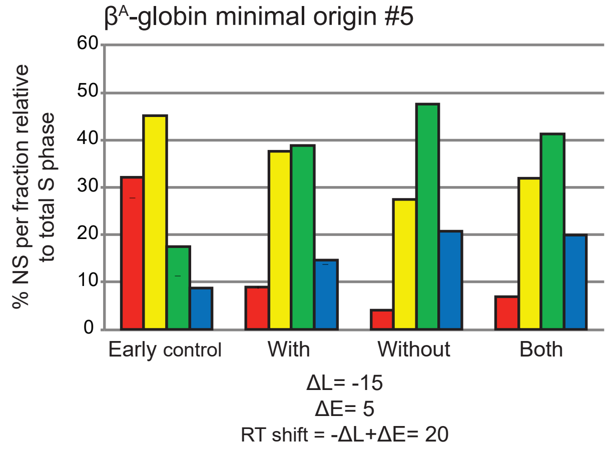

Supplementary Figure 6: RT analyses of the β^A-globin minimal origin

Scheme of the β^A-globin minimal origin coupled with the IL2R gene, SV40 PolyA sequence and USF binding sites is shown at the top. The corresponding sequence is given below each scheme with pG4s underlined and cis-regulatory elements delineated by colored boxes (green for the CCAAT-Box and brown for TATA-Box). Analyses of five β^A-globin minimal origin clonal cell lines are reported. RT profiles of each chromosomal allele is determined after targeted transgene integration using the allele-specific analysis of RT method by real-time PCR quantification as described in supplementary figure 2. Source data are provided as a Source Data file.

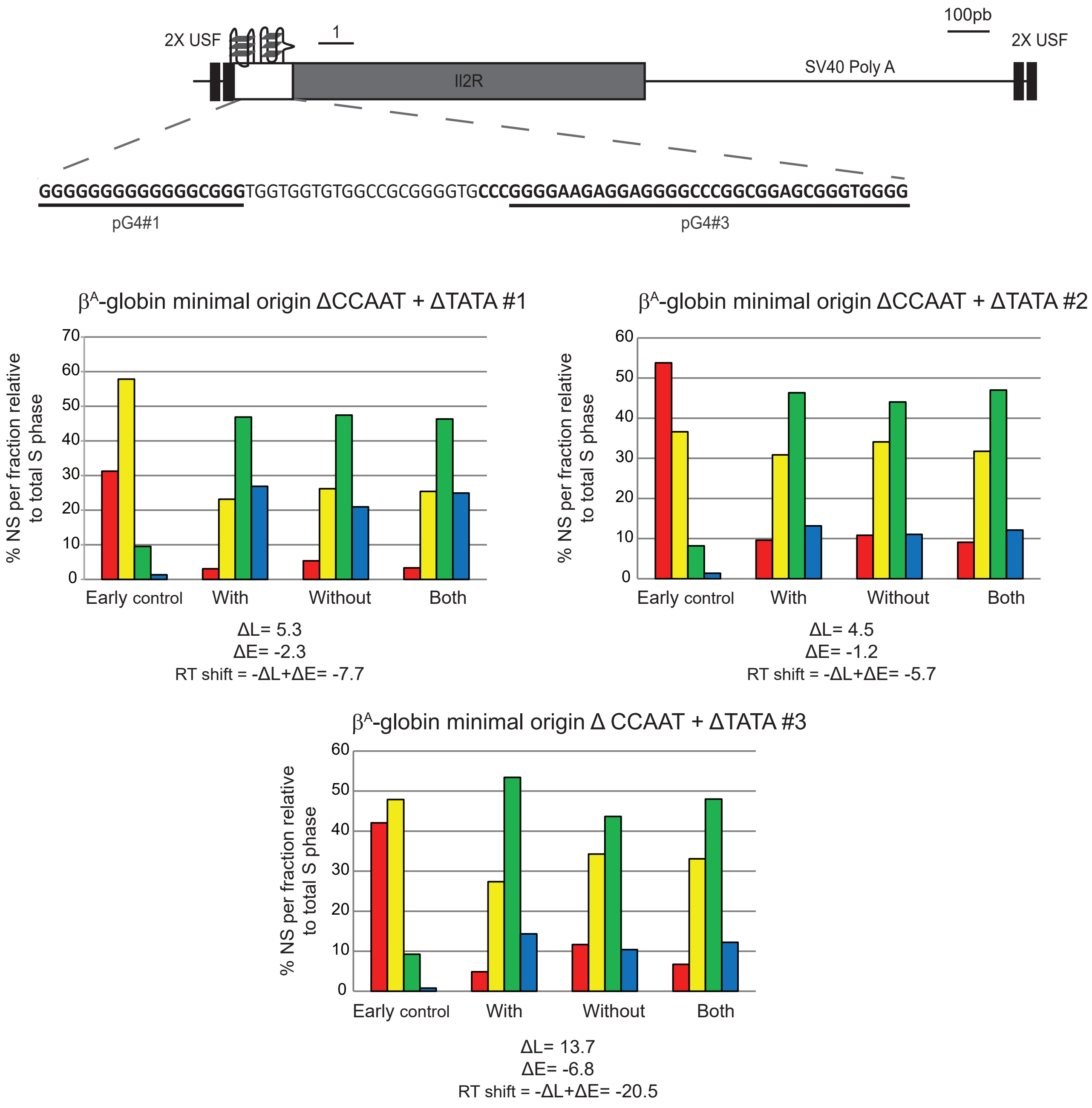

Supplementary Figure 7: RT analyses of the β^{A}-globin minimal origin ΔCCAAT+ΔTATA

Scheme of the β^{A}-globin minimal origin ΔCCAAT+ΔTATA coupled with the IL2R gene, SV40 PolyA sequence and USF binding sites is shown at the top. The corresponding sequence is given below with pG4s underlined. Analyses of three β^{A}-globin minimal origin ΔCCAAT+ΔTATA clonal cell lines are reported. RT profiles of each chromosomal allele is determined after targeted transgene integration using the allele-specific analysis of RT method by real-time PCR quantification as described in supplementary figure 2. Source data are provided as a Source Data file.

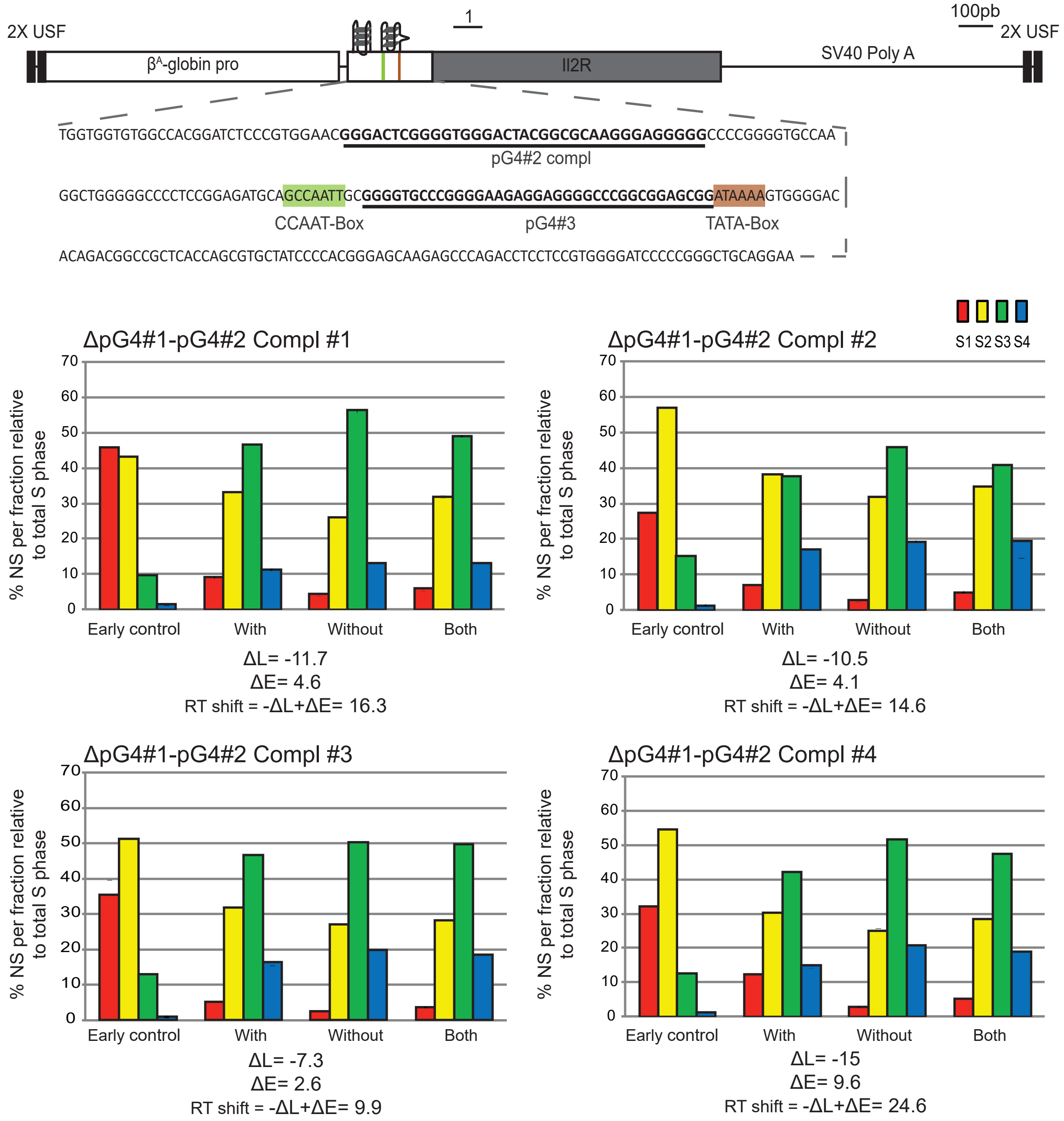

Supplementary Figure 8: RT analyses of the ΔpG4#1 pG4#2 Compl βA-globin origin
Scheme of the ΔpG4#1 pG4#2 Compl βA-globin origin coupled with the IL2R gene, SV40 PolyA sequence and USF binding sites is shown at the top. The corresponding sequence is given below with pG4s underlined and *cis*-regulatory elements delineated by colored boxes (green for the CCAAT-Box and brown for TATA-Box). Analyses of four ΔpG4#1 pG4#2 Compl βA-globin origin clonal cell lines are reported. RT profiles of each chromosomal allele is determined after targeted transgene integration using the allele-specific analysis of RT method by real-time PCR quantification as described in supplementary figure 2. Source data are provided as a Source Data file.

Synchronized β^{A}-globin minimal origin

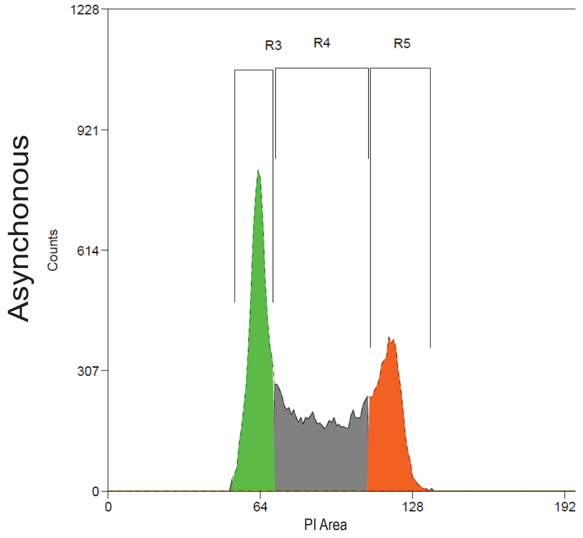

Region	Count	% Hist	Median	Mean	CV
Total	20000	100.00	83.00	86.83	26.34
R3	7151	35.75	63.00	62.82	5.62
R4	7789	38.95	88.00	88.80	13.68
R5	5024	25.12	118.00	118.07	4.03

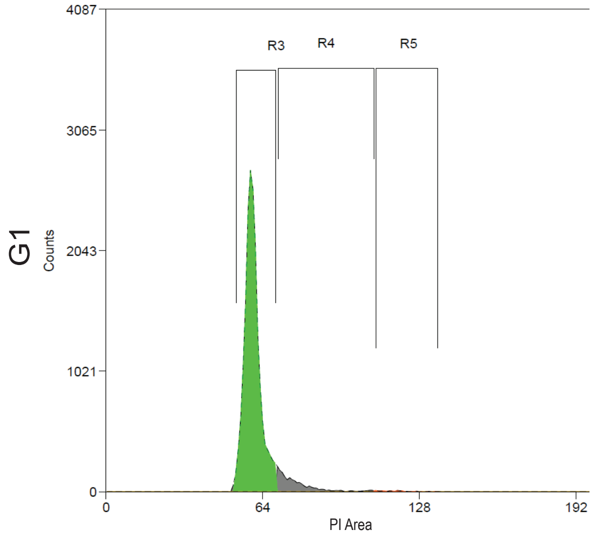

Region	Count	% Hist	Median	Mean	CV
Total	20000	100.00	60.00	61.83	12.92
R3	18009	90.05	60.00	59.84	5.29
R4	1801	9.01	75.00	77.97	10.95
R5	128	0.64	118.00	118.34	5.27

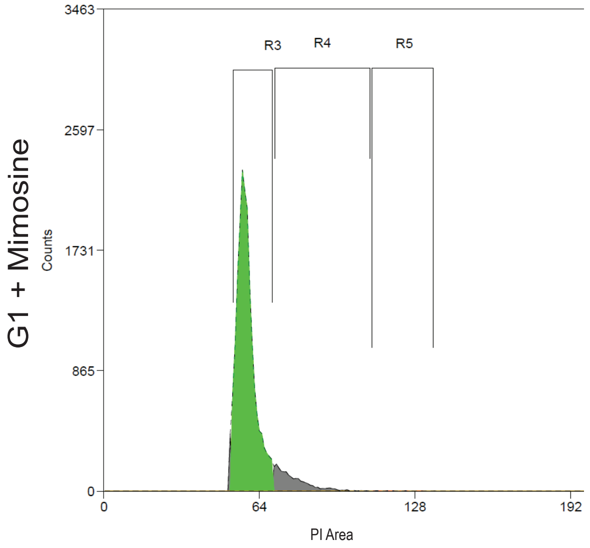

Region	Count	% Hist	Median	Mean	CV
Total	20000	100.00	58.00	60.55	12.62
R3	17523	87.61	58.00	58.61	6.13
R4	2033	10.17	76.00	77.99	9.37
R5	31	0.16	117.00	119.03	4.99

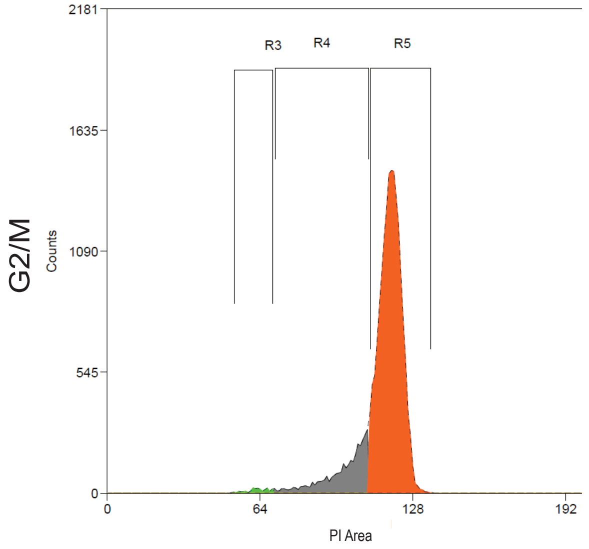

Region	Count	% Hist	Median	Mean	CV
Total	20000	100.00	118.00	114.84	9.33
R3	222	1.11	63.00	62.67	6.77
R4	3292	16.46	102.00	98.92	9.53
R5	16479	82.39	119.00	118.73	3.59

Supplementary Figure 9: Validation of the β^{A}-globin minimal origin cell synchronization by elutriation and L-mimosine incubation

Cell cycle analyses by flow cytometry for different fractions of elutriated cells containing the β^{A}-globin minimal origin were made after DNA labelling with propidium iodide (PI). The DNA content distributions of cells in G1-phase before (G1) and G1 plus 3 hrs of L-mimosine incubation (G1+Mimosine) or in G2/M-phase (G2/M) are shown. G1-phase cells are represented in green and G2/M cells in orange. R3, R4 and R5 regions were settled based on the asynchronous cell profile to define the proportion of cells in each fraction. Proportions of cells analyzed for each condition as well as statistics of the distribution of the detected PI values for each region are given in the table below each graph. Black titles on the Y-axis give the name of the cell population analyzed.

Synchronised β^{A}-globin minimal origin pG4#1 rev compl

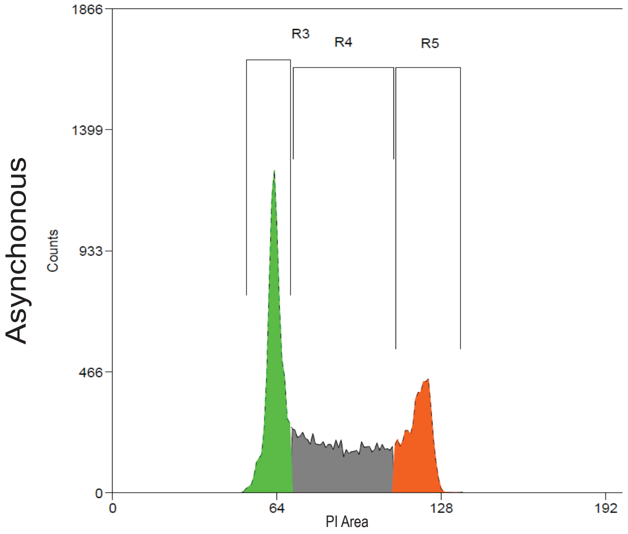

Region	Count	% Hist	Median	Mean	CV
Total	20000	100.00	80.00	86.08	26.74
R3	7704	38.52	63.00	63.02	4.78
R4	7393	36.96	88.00	88.43	13.36
R5	4896	24.48	119.00	118.82	3.76

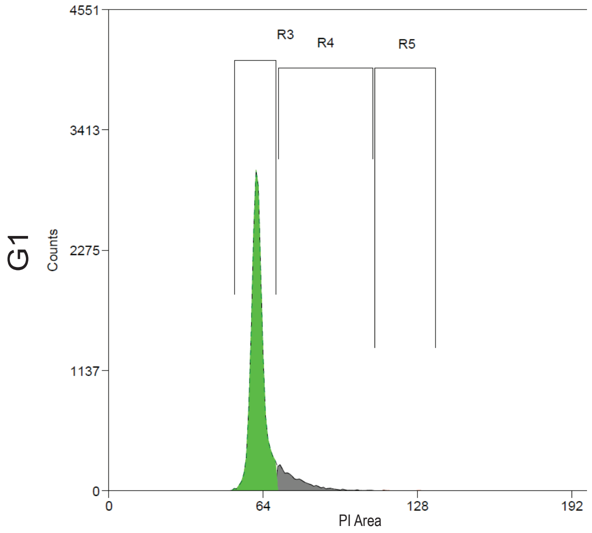

Region	Count	% Hist	Median	Mean	CV
Total	19189	100.00	62.00	63.75	10.35
R3	16785	87.47	62.00	61.71	4.31
R4	2379	12.40	76.00	77.65	9.31
R5	20	0.10	116.00	119.30	5.35

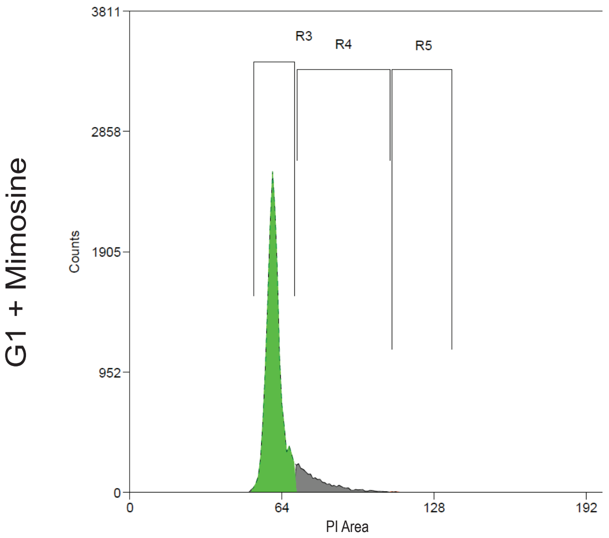

Region	Count	% Hist	Median	Mean	CV
Total	18386	100.00	61.00	63.26	12.47
R3	15790	85.88	60.00	60.64	5.06
R4	2554	13.89	77.00	79.13	10.62
R5	23	0.13	112.00	114.09	4.12

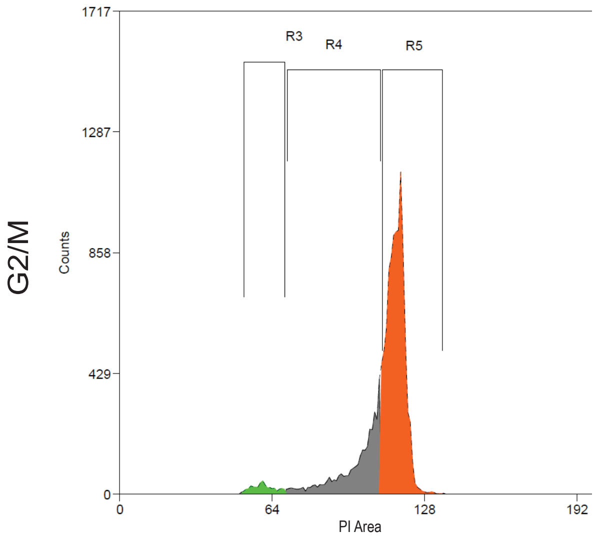

Region	Count	% Hist	Median	Mean	CV
Total	13150	100.00	114.00	109.93	11.82
R3	422	3.21	60.00	60.27	7.58
R4	3406	25.90	103.00	99.48	9.67
R5	9312	70.81	116.00	116.04	2.96

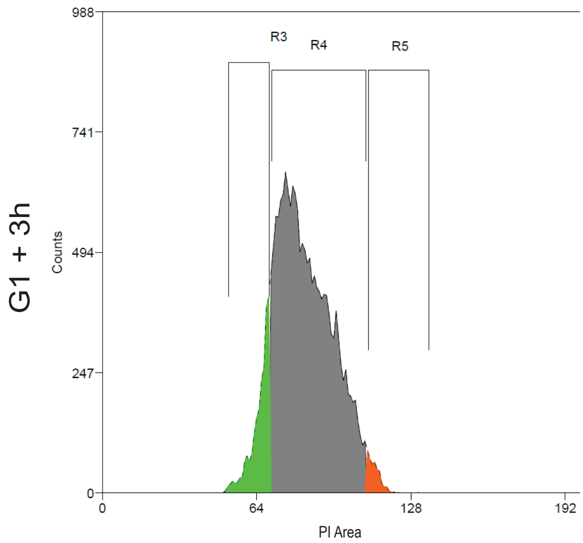

Region	Count	% Hist	Median	Mean	CV
Total	18685	100.00	82.00	83.42	14.74
R3	2161	11.57	66.00	65.16	5.96
R4	16093	86.13	84.00	85.10	11.90
R5	427	2.29	112.00	112.83	2.27

Supplementary Figure 10: Validation of cells containing the β^{A}-globin minimal inactive origin synchronization by elutriation and L-mimosine incubation

Cell cycle analyses by flow cytometry of different fractions of elutriated of cells containing the β^{A}-globin minimal origin pG4#1 reverse complement (β^{A}-globin minimal origin pG4#1 rev compl) were made as described in supplementary figure 9. G1-phase cells released for 3 hrs in normal culture conditions were also tested as control of the cell population viability after elutriation.

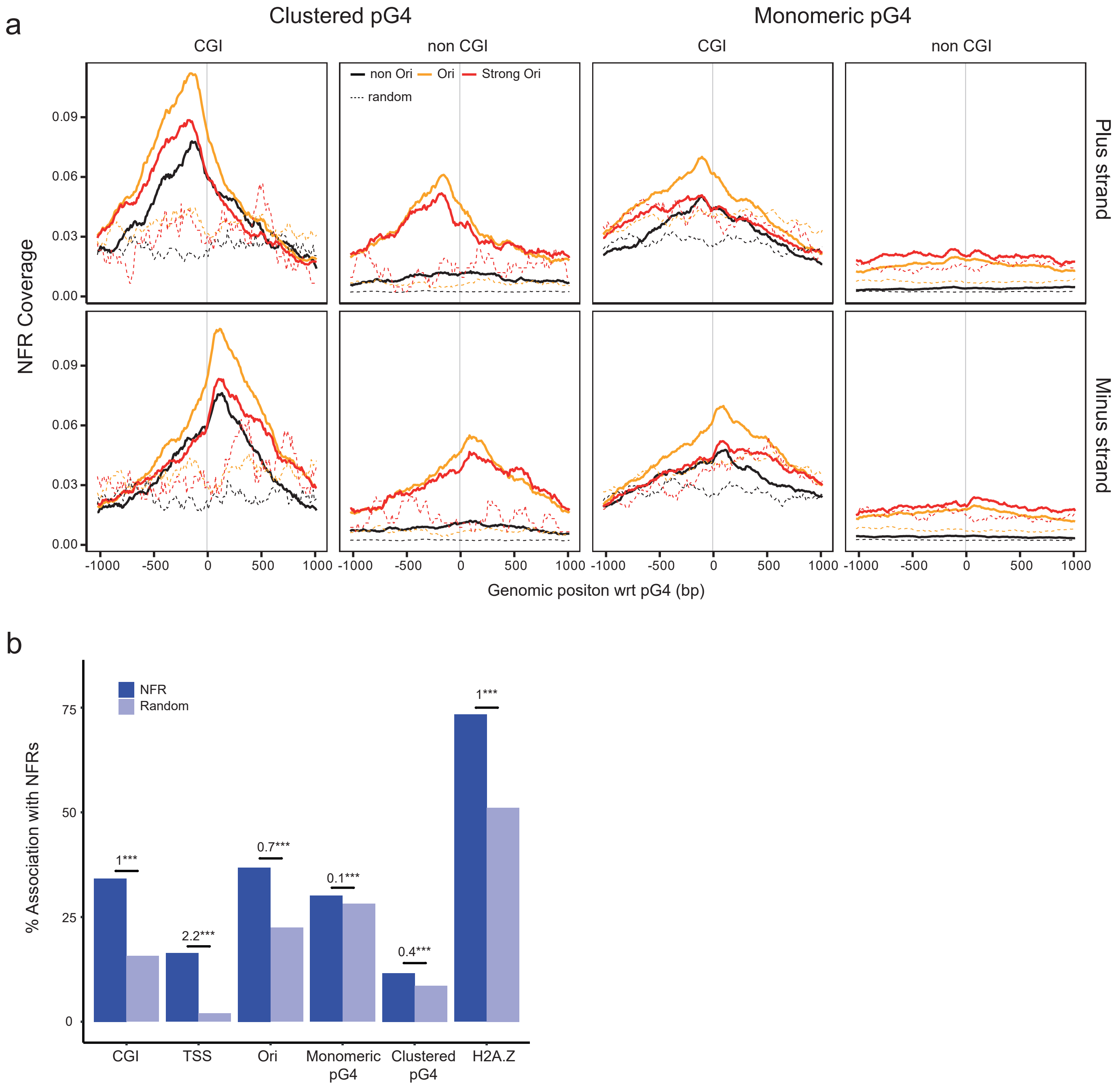

Supplementary Figure 11

(a) NFR coverage in G2/M cells around clustered pG4s (left) and monomeric pG4s (right) on the plus (top) and minus (bottom) strand. (b) Association of NFRs with genomic features in chicken cells. Numbers above bars indicate the log-odd-ratio of the logistic regression to test enrichment with respect to random segments. ***P < 0.001 by the Wald test (see methods).

Supplementary Figure 12

(a) Nucleosome coverage around cluster (left) and monomeric (right) pG4s on the minus strand. (b) NFR coverage in G1 cells around cluster (left) and monomeric (right) pG4s on the minus strand. (c) Short Nascent Strand coverage around cluster (left) and monomeric (right) pG4s on the minus strand. (d) Cluster (left) and monomeric (right) pG4s coverage around NFR centers on the minus strand. (e) H2A.Z coverage around origins' centers containing cluster (left) or monomeric (right) pG4s found on either the plus (top) or the minus (bottom) strand.

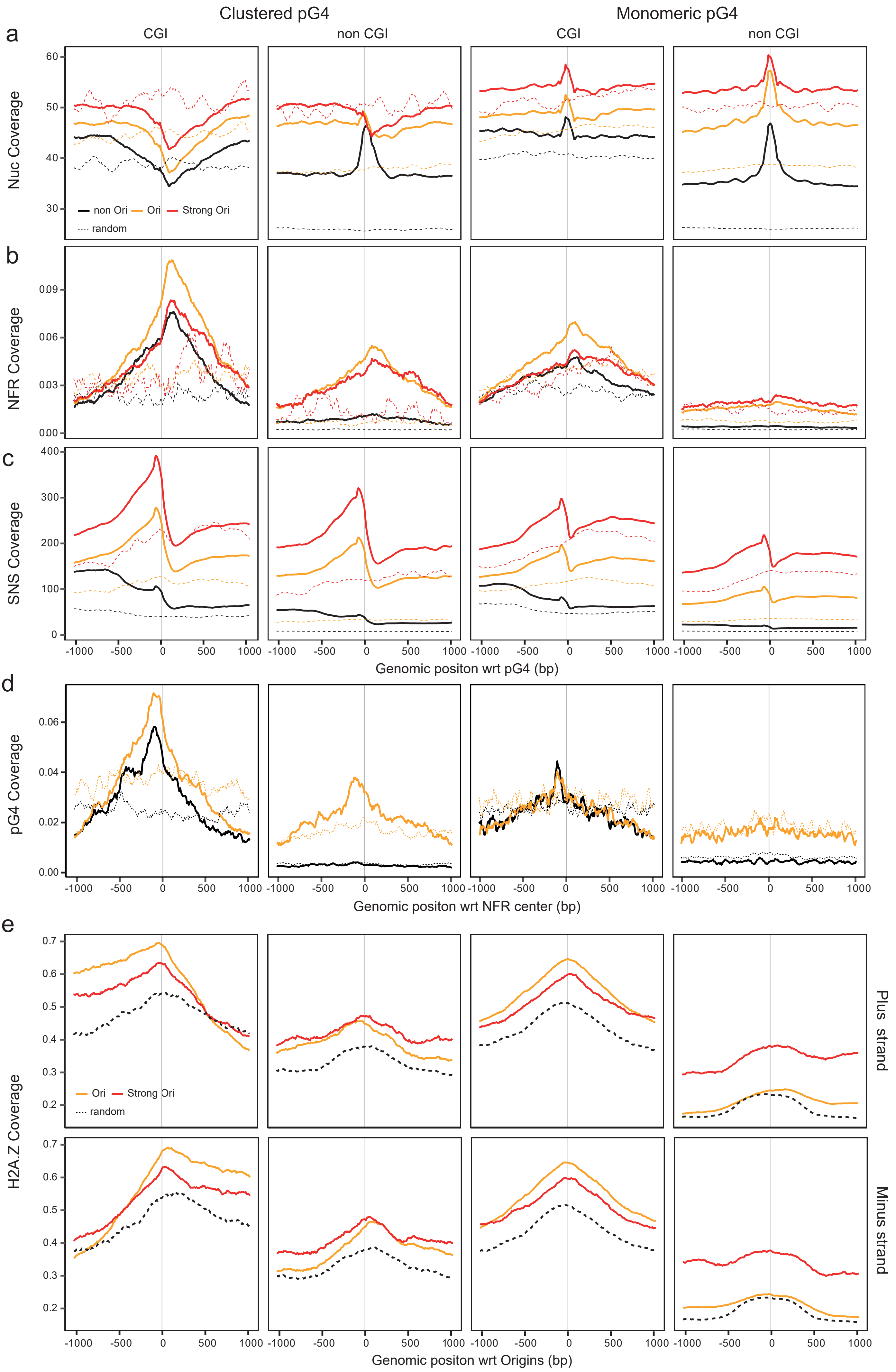

Clustered pG4
Monomeric pG4
CGI
non CGI
a
Nuc Coverage
non Ori
Ori
Strong Ori
random
b
NFR Coverage
c
SNS Coverage
Genomic positon wrt pG4 (bp)
d
pG4 Coverage
Genomic positon wrt NFR center (bp)
e
H2A.Z Coverage
Plus strand
Minus strand
Genomic positon wrt Origins (bp)

Synchronised wt DT40 cells

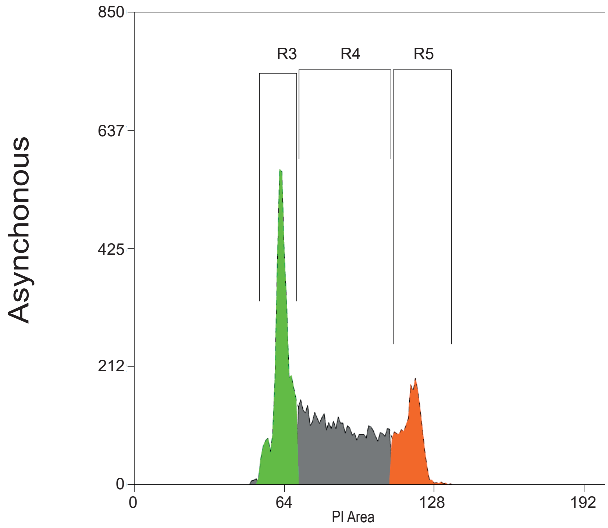

Region	Count	% Hist	Median	Mean	CV
Total	9752	100.00	79.00	84.27	25.48
R3	3611	37.03	63.00	62.85	5.28
R4	4258	43.66	87.00	87.97	13.31
R5	1857	19.04	118.00	117.87	3.64

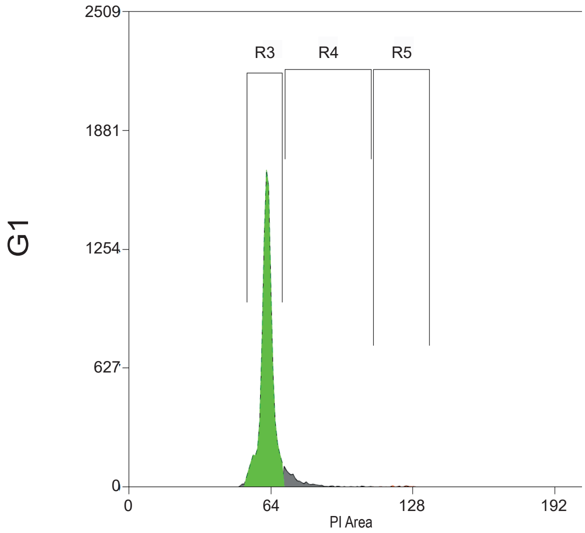

Region	Count	% Hist	Median	Mean	CV
Total	9909	100.00	62.00	63.46	10.44
R3	9100	91.84	62.00	62.15	4.54
R4	722	7.29	74.00	76.41	9.92
R5	50	0.50	122.00	122.08	4.65

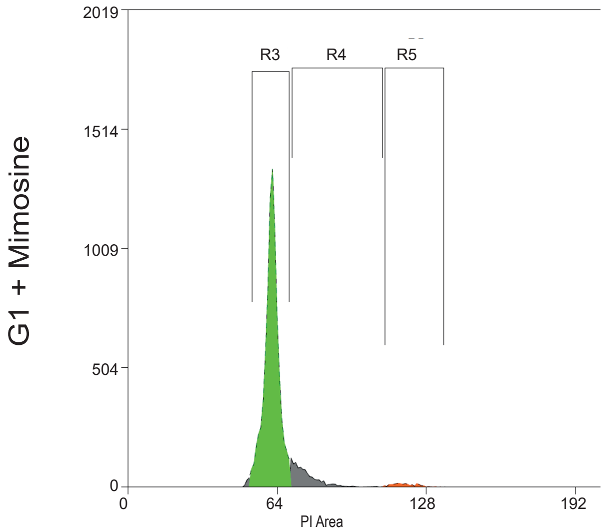

Region	Count	% Hist	Median	Mean	CV
Total	9517	100.00	62.00	64.27	15.39
R3	8215	86.32	62.00	61.57	5.02
R4	1033	10.85	75.00	76.61	8.62
R5	191	2.01	118.00	118.80	4.29

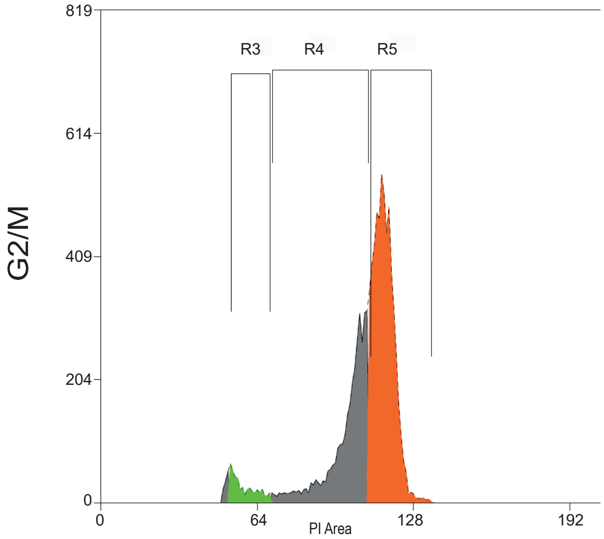

Region	Count	% Hist	Median	Mean	CV
Total	9527	100.00	112.00	107.09	14.70
R3	452	4.74	57.00	58.79	8.41
R4	3384	35.52	104.00	100.57	8.73
R5	5578	58.55	116.00	116.07	3.48

Supplementary Figure 13: Validation of the DT40 wt cell synchronization by elutriation for ATAC-Seq experiments
Cell cycle analyses by flow cytometry for different fractions of elutriated DT40 wt cells were made after DNA labelling with propidium iodide (PI). The DNA content distributions of cells in G1-phase before (G1) or in G2/M-phase (G2/M) are shown. G1-phase cells are represented in green and G2/M cells in orange. R3, R4 and R5 regions were settled based on the asynchronous cell profile to define the proportion of cells in each fraction. Proportions of cells analyzed for each condition as well as statistics of the distribution of the detected PI values for each region are given in the table below each graph. Black titles on the Y-axis give the name of the cell population analyzed.

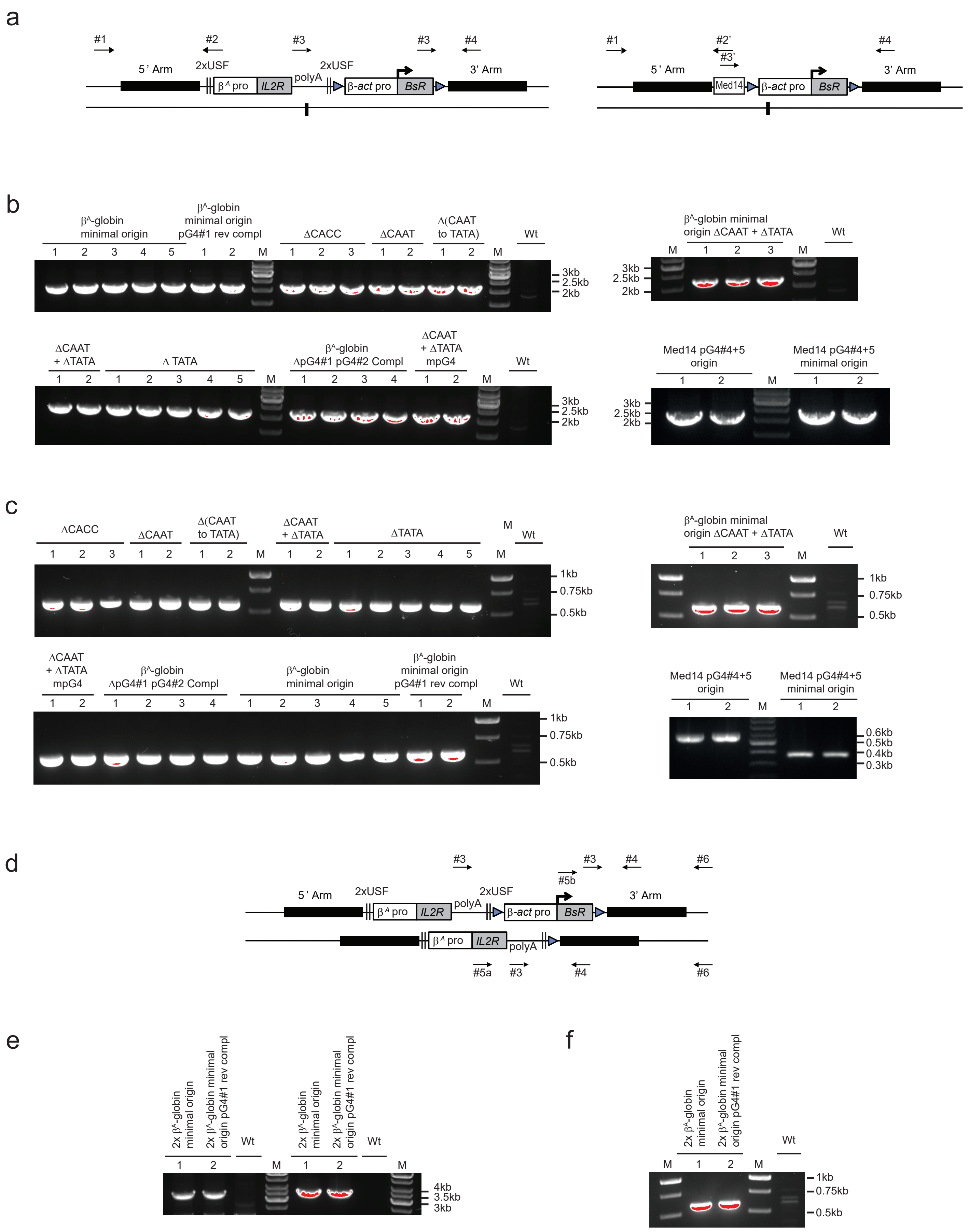

Supplementary Figure 14: PCR validation of clones selected for homologous recombination
Source data are provided at the end of the Supplementary Information file.

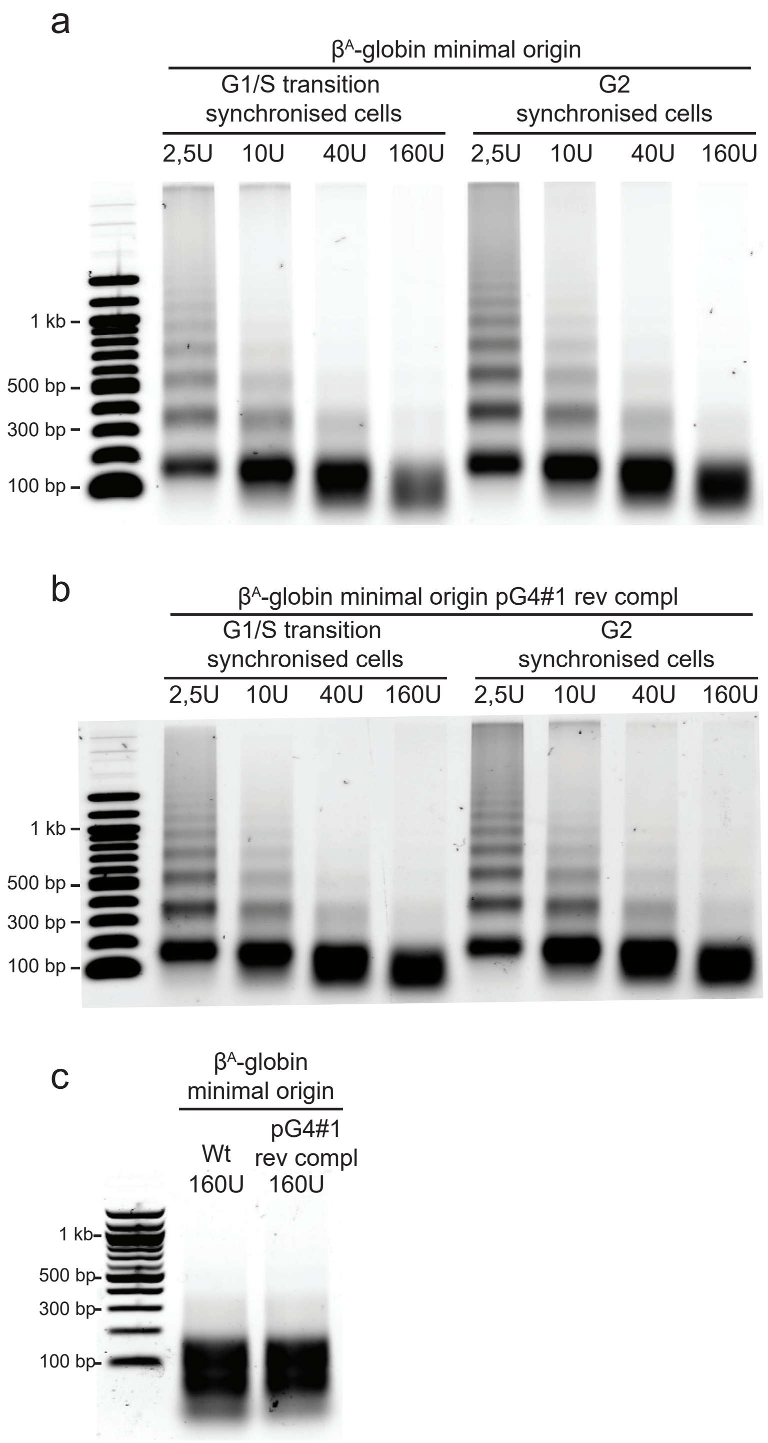

Supplementary Figure 15: Validation of MNase digestion patterns obtained to analyse nucleosome positioning
(a-c) Chromatin was extracted from clonal cell lines containing the β^A-globin minimal origin (a, c) or the β^A-globin minimal origin containing the pG4#1 reverse complementary sequence (pG4#1 rev compl, b, c). Chromatin was partially digested with exponentially increasing concentrations of micrococcal nuclease (MNAse; 2.5, 10, 40 and 160 U/mL). The four digested DNA samples obtained for each clonal cell line (a and b) or the most digested sample only (160U/mL, c) were subjected to electrophoresis in a 1% w/v agarose gel and stained with SYBR safe. The DNA size marker was a commercial 100bp ladder. (a-b) Digestion patterns of cells synchronised at the G1/S transition are shown on the left and those of cells in G2-phase are shown on the right. (c) Digestion patterns of asynchronous cells are shown. Source data are provided at the end of the Supplementary Information file.

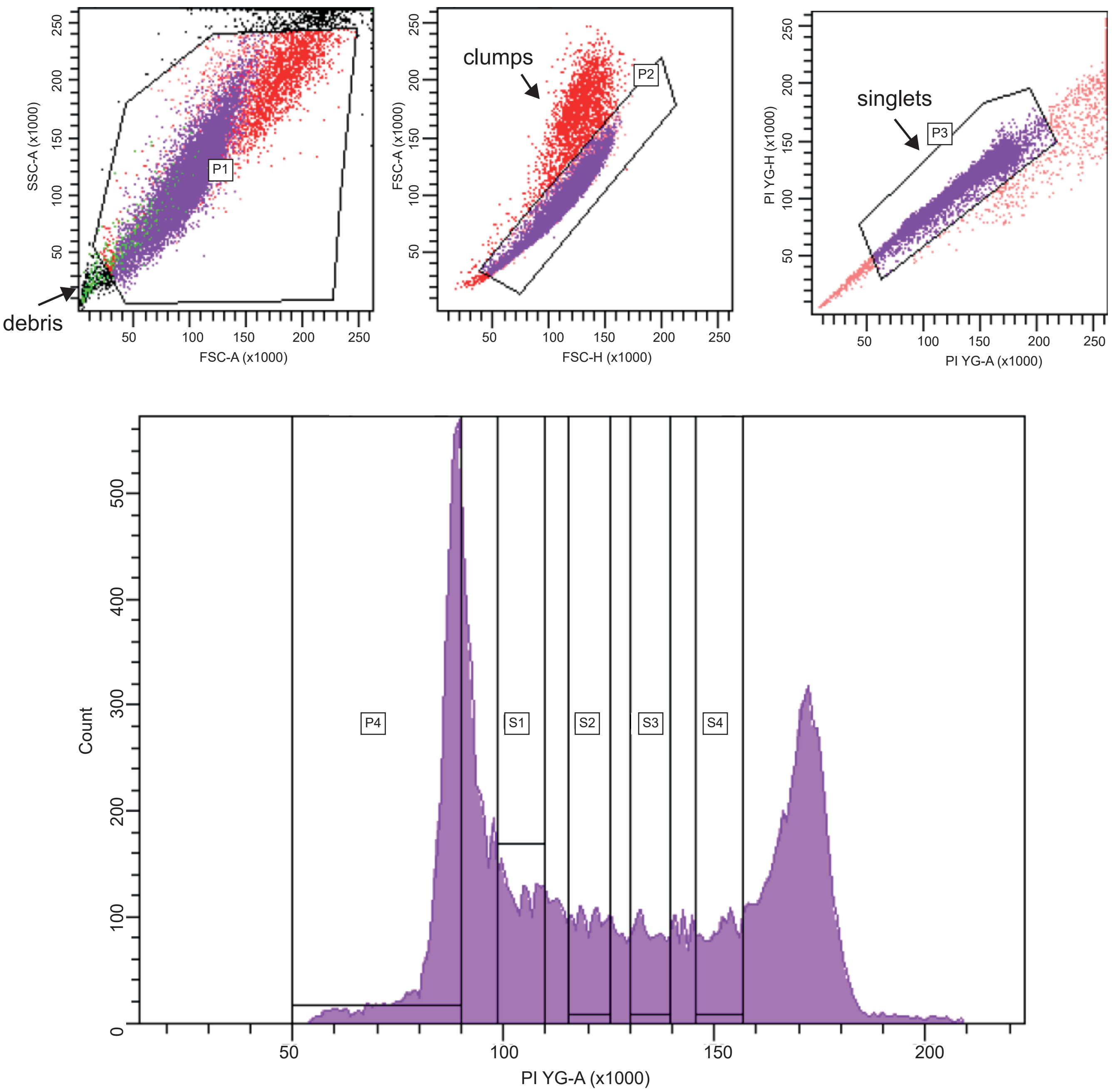

	#Events	%Parent	%Total
All Events	22,917		100.0
P1	19,317	84.3	84.3
P2	16,734	86.6	73
P3	15,696	93.8	8.5
P4	3,211	20.5	14
S1	1,393	8.9	6.1
S2	941	6.0	4.1
S3	38	5.3	3.7
S4	993	6.3	4.3
	291	1.3	1.3

Supplementary Figure 16: Gating strategy for cell sorting in RT analyses
Cell sorting by flow cytometry was made after DNA labelling with propidium iodide (PI). The whole cell population was selected on the dot diagram of side scatter (SSC) versus forward scatter (FSC) (P1). Cell debris and clumps were removed successively on dot diagrams of FSC-A versus FSC-H (P2) and PI-H versus PI-A (P3). The PI fluorescent signal was detected by the yellow-green channel (YG). P3 gate derived successively form P2 and P1 correspond to singlets cells. Voltage (PMT) is adjusted to position the G1 peak at the same fluorescence intensity for all clones analyed. S1 to S4 fractions were defined as regions of equal width inside the S-phase profile and separated equally. Number of events analyzed (#Events), proportion of cells in each region from the previous gate (%Parent) or from the total population (%Total) are given in the table below the graph.

Contents

1 Logistic regression to quantify genome-wide associations 2

2 How to assess if Origins are enriched in Monomeric or in Clustered pG4s ? 2

List of Tables

1 Association of pG4s with genomic features in human with SNS Oris 4
2 Association of pG4s with genomic features in chicken and mouse 5
3 Association of origins in genomic features in human 6
4 Association of origins in genomic features in chicken and mouse 7
5 Association of strong origins in genomic features in human 8
6 Association of strong origins in genomic features in chicken and mouse 9
7 Associations of H2AZ in genomic features in human, chicken and mouse . . . 10
8 Associations of NFRs in genomic features in chicken 11

1 Logistic regression to quantify genome-wide associations

Here we provide some details regarding the statistical analysis of associations between genomic features. Considering an example with the association of origins with monomeric pG4s (from Supp Table), as illustrated in the contingency table / odds ratio

All Oris	#inOri	#Ori	%inOri	#inRand	#Rand	%inRand	LogOR
Monomeric pG4	140,527	310,790	45	90,965	306,620	30	0.7

This table contains the probabilities of association that provide the chance of an event happening. If we consider the variable Ori = 1 when a given segment is a replication origins, and Ori = 0 is the segment is a random segment, we can also compute the odds such that:

$$\begin{aligned}
\text{Odd of an origin carrying a Mono pG4} &= \frac{140,527}{310,790 - 140,527} = 0.8253525 \\
\text{Odd of a random segment carrying a Mono pG4} &= \frac{90,965}{306,620 - 90,965} = 0.421808
\end{aligned}$$

Odds are the ratio of an association vs. non association

$$\text{Odd} = \frac{p}{1-p} = \frac{\text{probability of success}}{\text{probability of failure}}$$

The interest of log-Odds is that they can be used to compare enrichments, for instance here, the enrichment in CGI for origins or for random segments:

$$\begin{aligned}
\log \text{Odd}(\text{Ori} = 1, \text{Mono pG4} = 1) &= -0.1919447 \\
\log \text{Odd}(\text{Ori} = 0, \text{Mono pG4} = 1) &= -0.8632051
\end{aligned}$$

Here, log-Odds ratio becomes:

$$\log \text{Odd}(\text{Ori} = 1, \text{Mono pG4} = 1) - \log \text{Odd}(\text{Ori} = 0, \text{Mono pG4} = 1) = 0.6712604 > 0$$

Since the logOR is positive, it means that origins are more enriched in Monomeric pG4s than random segments.

This general framework is the logistic regression developed to quantify and test associations between a binary variable and covariates. We used the `emmeans` `R`-package (version 1.7.3) for computing logistic regressions. In this framework, the significance of log-odds ratios is assessed using z-tests.

2 How to assess if Origins are enriched in Monomeric or in Clustered pG4s ?

In a second step we question whether origins are rather associated with the Monomeric or Clustered Form of pG4s. For this purpose we need to restrict the set of considered origins to origins that actually carry a pG4. Similarly, as a control, we consider random segments that carry a pG4. Then we ask if when origins carry a pG4, they are more enriched in Monomeric pG4 (for instance). Considering the table :

Oris with pG4	#inOri	#Ori	%inOri	#inRand	#Rand	%inRand	LogOR
Monomeric pG4	140,527	166,257	84	90,965	103,032	88	-0.3***

The computed odds are:

$$\text{Odd of an origin that carries a pG4 of carrying a Mono pG4} = \frac{140,527}{166,257 - 140,527} = 5.461601$$

$$\text{Odd of a random segment that carries a pG4 of carrying a Mono pG4} = \frac{90,965}{103,032 - 90,965} = 7.538328$$

Then the log-Odds ratio becomes:

$$\log \text{Odd}(\text{Ori} = 1, \text{Mono pG4} = 1 \mid \text{pG4} = 1) - \log \text{Odd}(\text{Ori} = 0, \text{Mono pG4} = 1 \mid \text{pG4} = 1) = -0.3222584,$$

with notation $\mid \text{pG4} = 1$ meaning that the analysis is restricted to segments that carry pG4s. Very interestingly, this logOR has become negative ! meaning that when we restrict the enrichment analysis to segments that carry a pG4, the replication origins are not enriched in the Monomeric form of pG4s, but rather in the Clustered form (logOR=0.5 in Supp. Table). A similar strategy was used to assess the association of H2AZ and NFRs with pG4s.

pG4 Human							
**Monomeric pG4**	#inpG4	#pG4	%inpG4	#inRand	#Rand	%inRand	LogOR
CGI	105693	1035770	10	23836	1035770	2	1.6***
TSS	57046	1035770	6	14442	1035770	1	1.4***
Oris H9 SNS							
Ori	275440	1035770	27	92948	1035770	9	1.3***
Strong Ori	115157	1035770	11	23479	1035770	2	1.7***
H2AZ	433256	1035770	42	374299	1035770	36	0.2***
H2AZ in pG4 with Ori	136311	275440	50	46014	92948	50	0^{ns}
H2AZ in pG4 without Ori	296945	760330	39	328285	942822	35	0.2***
chIP G4, Zheng et al. 2020	418938	1035770	40	153749	1035770	15	1.4***
chIP G4 in pG4 with Ori	195993	275440	71	47163	92948	51	0.9***
chIP G4 in pG4 without Ori	222945	760330	29	106586	942822	11	1.2***
chIP G4, Hansel-Hertsch et al. 2016	21599	1035770	2	7020	1035770	1	1.1***
chIP G4 with Ori	12246	275440	4	2736	93023	3	0.4***
chIP G4 without Ori	9353	760330	1	4284	942747	0	1***
Core Oris from Akerman et al. (2020), SNS							
Ori	131148	1035770	13	35900	1035770	4	1.4***
Oris EJ30 from Guilbaud et al. (2022), Iniseq2							
Ori	35706	1035770	3	15335	1035770	2	0.9***
Strong Oris	7343	1035770	1	4002	1035770	0	0.6***
**Clustered pG4**	#inpG4	#pG4	%inpG4	#inRand	#Rand	%inRand	LogOR
CGI	37600	156441	24	3392	156441	2	2.7***
TSS	21585	156441	14	2130	156441	1	2.5***
Oris H9 SNS							
Ori	73581	156441	47	13646	156441	9	2.2***
Strong Ori	42713	156441	27	3367	156441	2	2.8***
H2AZ	67792	156441	43	56299	156441	36	0.3***
H2AZ in pG4 with Ori	37439	73581	51	6706	13646	49	0.1***
H2AZ in pG4 without Ori	30353	82860	37	49593	142795	35	0.1***
chIP G4, Zheng et al. 2020	106590	156441	68	22782	156441	15	2.5***
chIP G4 in pG4 with Ori	62188	73581	84	6801	13646	50	1.7***
chIP G4 in pG4 without Ori	44402	82860	54	15981	142795	11	2.2***
chIP G4, Hansel-Hertsch et al. 2016	7042	156441	4	1034	156441	1	2***
chIP G4 with Ori	4966	73581	7	383	13676	3	0.9***
chIP G4 without Ori	2076	82860	2	651	142765	0	1.7***
Core Oris from Akerman et al. (2020), SNS							
Ori	50905	156441	32	5265	156441	3	2.6***
Oris EJ30 from Guilbaud et al. (2022), Iniseq2							
Ori	9542	156441	6	2219	156441	1	1.5***
Strong Oris	1859	156441	1	579	156441	0	1.2***

Table 1: Association of pG4s with genomic features. Associations were computed separately for pG4+ and pG4− and combined. Strong Oris are the top 25% active origins in terms of SNS enrichment. Random pG4 segments were sampled to match the length distribution of observed pG4s (see Material & Methods). #inpG4: number of features in pG4, #pG4: number of pG4s, %inpG4: percentage of features in pG4, #inRand: number of features in random segments, #Rand: number of random segments, %inRand: percentage of feature in random segments, LogOR: log-odd-ratio of the logistic regression to test enrichment in pG4 wrt enrichment in random segments (see Material & Methods).

pG4 Chicken (DT40)							
Monomeric pG4	#inpG4	#pG4	%inpG4	#inRand	#Rand	%inRand	LogOR
CGI	67003	300974	22	13581	300974	4	1.8***
TSS	15833	300974	5	3276	300974	1	1.6***
Ori	92201	300974	31	35127	300974	12	1.2***
Strong Oris	47142	300974	16	8866	300974	3	1.8***
H2AZ	196050	300974	65	122209	300974	41	1***
H2AZ in pG4 with Ori	73530	92201	80	23255	35127	66	0.7***
H2AZ in pG4 without Ori	122520	208773	59	98954	265847	37	0.9***
NFR in G1	28394	300974	9	11446	300974	4	1***
NFR in G1 in pG4 with Ori	16279	92201	18	3711	35127	11	0.6***
NFR in G1 in pG4 without Ori	12115	208773	6	7735	265847	3	0.7***
NFR in G2	23382	300974	8	8272	300974	3	1.1***
NFR in G2 in pG4 with Ori	14334	92201	16	3115	35127	9	0.6***
NFR in G2 in pG4 without Ori	9048	208773	4	5157	265847	2	0.8***
chIP G4, Zheng et al. 2020	44677	300974	15	14078	300974	5	1.3***
chIP G4 in pG4 with Ori	26074	92201	28	5565	35127	16	0.7***
chIP G4 in pG4 without Ori	18603	208773	9	8513	265847	3	1.1***
Clustered pG4	#inpG4	#pG4	%inpG4	#inRand	#Rand	%inRand	LogOR
CGI	24593	53575	46	2271	53575	4	3***
TSS	7158	53575	13	496	53575	1	2.8***
Ori	27745	53575	52	6279	53575	12	2.1***
Strong Oris	18425	53575	34	1523	53575	3	2.9***
H2AZ	42468	53575	79	21595	53575	40	1.7***
H2AZ in pG4 with Ori	23927	27745	86	4069	6279	65	1.2***
H2AZ in pG4 without Ori	18541	25830	72	17526	47296	37	1.5***
NFR in G1	10181	53575	19	2019	53575	4	1.8***
NFR in G1 in pG4 with Ori	7186	27745	26	641	6279	10	1.1***
NFR in G1 in pG4 without Ori	2995	25830	12	1378	47296	3	1.5***
NFR in G2	9265	53575	17	1395	53575	3	2.1***
NFR in G2 in pG4 with Ori	6710	27745	24	523	6279	8	1.3***
NFR in G2 in pG4 without Ori	2555	25830	10	872	47296	2	1.8***
chIP G4, Zheng et al. 2020	16491	53575	31	2450	53575	5	2.2***
chIP G4 in pG4 with Ori	11444	27745	41	995	6279	16	1.3***
chIP G4 in pG4 without Ori	5047	25830	20	1455	47296	3	2***

pG4 Mouse (mESC)							
Monomeric pG4	#inpG4	#pG4	%inpG4	#inRand	#Rand	%inRand	LogOR
CGI	51285	996347	5	14481	996347	2	1.3***
TSS	48146	996347	5	15619	996347	2	1.2***
Ori	262428	996347	26	137003	996347	14	0.8***
Strong Oris	105252	996347	11	35297	996347	4	1.2***
H2AZ	132367	996347	13	49792	996347	5	1.1***
H2AZ in pG4 with Ori	77739	262428	30	23967	137003	18	0.7***
H2AZ in pG4 without Ori	54628	733919	7	25825	859344	3	1***
chIP G4, Zheng et al. 2020	70078	996347	7	23524	996347	2	1.1***
chIP G4 in pG4 with Ori	48193	262428	18	13195	137003	10	0.7***
chIP G4 in pG4 without Ori	21885	733919	3	10329	859344	1	0.9***
Clustered pG4	#inpG4	#pG4	%inpG4	#inRand	#Rand	%inRand	LogOR
CGI	15610	156571	10	2161	156571	1	2.1***
TSS	14005	156571	9	2343	156571	2	1.9***
Ori	54108	156571	35	21070	156571	14	1.2***
Strong Oris	25881	156571	16	5337	156571	3	1.7***
H2AZ	26100	156571	17	7711	156571	5	1.4***
H2AZ in pG4 with Ori	7777	102463	8	4036	135501	3	1***
H2AZ in pG4 without Ori	18323	54108	34	3675	21070	17	0.9***
chIP G4, Zheng et al. 2020	19037	156571	12	3635	156571	2	1.8***
chIP G4 in pG4 with Ori	14931	54108	28	1977	21070	9	1.3***
chIP G4 in pG4 without Ori	4106	102463	4	1658	135501	1	1.2***

Table 2: Association of pG4s with genomic features. Associations were computed separately for pG4+ and pG4− and combined. Strong Oris are the top 25% active origins in terms of SNS enrichment. Random pG4 segments were sampled to match the length distribution of observed pG4s (see Material & Methods). #inpG4: number of features in pG4, #pG4: number of pG4s, %inpG4: percentage of features in pG4, #inRand: number of features in random segments, #Rand: number of random segments, %inRand: percentage of feature in random segments, LogOR: log-odd-ratio of the logistic regression to test enrichment in pG4 wrt enrichment in random segments (see Material & Methods).

Oris Human H9 SNS (155,395 Oris)							
All Oris	#inOri	#Ori	%inOri	#inRand	#Rand	%inRand	LogOR
CGI	47,814	310,790	15	16,237	290,194	6	1.1***
TSS	26,774	310,790	9	7,189	290,194	2	1.3***
all pG4s	166,257	310,790	54	90,544	290,194	31	0.9***
Monomeric pG4s	140,527	310,790	45	80,064	290,194	28	0.8***
Clustered pG4s	53,304	310,790	17	17,958	290,194	6	1.1***
H2AZ	72,530	310,790	23	39,193	290,194	14	0.6***
chIP G4, Zheng et al. 2020	166,857	310,790	54	103,615	290,194	36	0.7***
chIP G4, Hansel-Hertsch et al. 2016	9,925	310,790	3	3,466	289,256	1	1***
Oris with pG4	#inOri	#Ori	%inOri	#inRand	#Rand	%inRand	LogOR
CGI	35,941	166,257	22	9,435	90,544	10	0.9***
TSS	20,578	166,257	12	4,022	90,544	4	1.1***
Monomeric pG4s	140,527	166,257	84	80,064	90,544	88	-0.3***
Clustered pG4s	53,304	166,257	32	17,958	90,544	20	0.6***
H2AZ	42,172	166,257	25	13,176	90,544	15	0.7***
chIP G4, Zheng et al. 2020	110,597	166,257	66	49,910	90,544	55	0.5***
chIP G4, Hansel-Hertsch et al. 2016	7,136	166,257	4	1,662	90,208	2	0.9***

Oris Human Core from Akerman et al. (2020), SNS, (63,624 Oris)							
All Oris	#inOri	#Ori	%inOri	#inRand	#Rand	%inRand	LogOR
CGI	44,494	127,248	35	4,563	127,248	4	2.7***
TSS	18,843	127,248	15	2,869	127,248	2	2***
all pG4s	74,716	127,248	59	33,201	127,248	26	1.4***
Monomeric pG4s	59,854	127,248	47	29,629	127,248	23	1.1***
Clustered pG4s	33,293	127,248	26	6,350	127,248	5	1.9***
chIP G4, Zheng et al. 2020	83,066	127,248	65	36,087	127,248	28	1.6***
chIP G4, Hansel-Hertsch et al. 2016	6,036	127,248	5	1,423	127,248	1	1.5***
Oris with pG4	#inOri	#Ori	%inOri	#inRand	#Rand	%inRand	LogOR
CGI	32,835	74,716	44	2,729	33,201	8	2.2***
TSS	15,412	74,716	21	1,666	33,201	5	1.6***
Monomeric pG4s	59,854	74,716	80	29,629	33,201	89	-0.7***
Clustered pG4s	33,293	74,716	45	6,350	33,201	19	1.2***
chIP G4, Zheng et al. 2020	58,259	74,716	78	17,723	33,201	53	1.1***
chIP G4, Hansel-Hertsch et al. 2016	4,675	74,716	6	726	33,339	2	1.1***

Oris Human EJ30 from Guilbaud et al. (2022), Iniseq2 (23,817 Oris)							
All Oris	#inOri	#Ori	%inOri	#inRand	#Rand	%inRand	LogOR
CGI	30,936	47,634	65	431	47,634	1	5.3***
TSS	20,390	47,634	43	358	47,634	1	4.6***
all pG4s	36,827	47,634	77	6,864	47,634	14	3***
Monomeric pG4s	29,693	47,634	62	6,344	47,634	13	2.4***
Clustered pG4s	16,396	47,634	34	716	47,634	2	3.5***
chIP G4, Zheng et al. 2020	45,763	47,634	96	5,776	47,634	12	5.2***
chIP G4, Hansel-Hertsch et al. 2016	7,967	47,634	17	216	47,634	0	3.8***
Oris with pG4	#inOri	#Ori	%inOri	#inRand	#Rand	%inRand	LogOR
CGI	24,316	36,827	66	174	6,864	2	4.3***
TSS	15,736	36,827	43	108	6,864	2	3.8***
Monomeric pG4s	29,693	36,827	81	6,344	6,864	92	-1.1***
Clustered pG4s	16,396	36,827	44	716	6,864	10	1.9***
chIP G4, Zheng et al. 2020	35,718	36,827	97	1,655	6,864	24	4.6***
chIP G4, Hansel-Hertsch et al. 2016	5,735	36,827	16	66	6,983	1	3***

Table 3: Enrichment of Origins in genomic features in human cells. Associations were computed separately 1kb downstream (pG4 on the minus strand) and 1 kb upstream the ori peak (pG4 on the plus strand), then combined so the total number of origins corresponds to 2 times the initial number indicated in the table's header. For instance, 155,395 Oris in the Massip et al. data, corresponding to 310,790 origins when combining + and − strand analyses (same for the random segments). Oris with pG4s consist in origins that carry a pG4. #inOri: number of features in Oris, #Ori: number of Oris, %inOri: percent of features in Oris, #inRand: number of features in random segments, #Rand: number of random segments, %inRand: percent of features in random segments, LogOR: log-odd-ratio of the logistic regression to test enrichment in Oris with respect to enrichment in random segments (see Material & Methods). When considering origins with pG4s, we restrict the analysis to segments that carry a pG4 (Oris and Random). By doing so we test whether, when origins or random segments are associated with a pG4, the enrichment concerns the monomeric or the Clustered form.

Oris Chicken DT40, SNS (68,068 Oris)							
All Oris	#inOri	#Ori	%inOri	#inRand	#Rand	%inRand	LogOR
CGI	33,233	136,136	24	16,104	126,356	13	0.8***
TSS	8,681	136,136	6	2,180	126,356	2	1.4***
all pG4s	57,209	136,136	42	40,495	126,356	32	0.4***
Monomeric pG4s	48,259	136,136	35	35,981	126,356	28	0.3***
Clustered pG4s	19,412	136,136	14	8,868	126,610	7	0.8***
H2AZ	84,841	136,136	62	67,347	126,356	53	0.4***
NFR in G1	16,035	136,136	12	6,626	126,356	5	0.9***
NFR in G2	13,560	136,136	10	4,421	126,356	4	1.1***
chIP G4, Zheng et al. 2020	24,176	136,136	18	9,321	126,356	7	1***
Oris with pG4	#inOri	#Ori	%inOri	#inRand	#Rand	%inRand	LogOR
CGI	22,736	57,209	40	10,125	40,495	25	0.7***
TSS	6,480	57,209	11	1,508	40,495	4	1.2***
Monomeric pG4s	48,259	57,209	84	35,981	40,495	89	-0.4***
Clustered pG4s	19,412	57,209	34	8,694	40,495	22	0.6***
H2AZ	41,959	57,209	73	26,393	40,495	65	0.4***
NFR in G1	10,049	57,209	18	3,240	40,495	8	0.9***
NFR in G2	8,960	57,209	16	2,234	40,495	6	1.2***
chIP G4, Zheng et al. 2020	15,669	57,209	27	4,937	40,495	12	1***

Oris Mouse mESC, SNS (205,881 Oris)							
All Oris	#inOri	#Ori	%inOri	#inRand	#Rand	%inRand	LogOR
CGI	36,889	411,762	9	2,294	190,458	1	2.1***
TSS	32,713	411,762	8	3,376	190,458	2	1.6***
all pG4s	176,495	411,762	43	56,545	190,458	30	0.6***
Monomeric pG4s	147,902	411,762	36	50,524	190,458	26	0.4***
Clustered pG4s	44,206	411,762	11	8,257	190,458	4	1***
H2AZ	80,454	411,762	20	21,795	190,458	11	0.6***
chIP G4, Zheng et al. 2020	45,480	411,762	11	7,015	190,458	4	1.2***
Oris with pG4	#inOri	#Ori	%inOri	#inRand	#Rand	%inRand	LogOR
CGI	24,309	176,495	14	1,123	56,545	2	2.1***
TSS	21,383	176,495	12	1,541	56,545	3	1.6***
Monomeric pG4s	147,902	176,495	84	50,524	56,545	89	-0.5***
Clustered pG4s	44,206	176,495	25	8,257	56,545	15	0.7***
H2AZ	46,300	176,495	26	8,894	56,545	16	0.6***
chIP G4, Zheng et al. 2020	29,084	176,495	16	3,054	56,545	5	1.2***

Table 4: Enrichment of Origins in genomic features in chicken and mouse cells. Associations were computed separately 1kb downstream (pG4 on the minus strand) and 1 kb upstream the ori peak (pG4 on the plus strand), then combined so the total number of origins corresponds to 2 times the initial number indicated in the table's header. Oris with pG4s consist in origins that carry a pG4. #inOri: number of features in Oris, #Ori: number of Oris, %inOri: percent of features in Oris, #inRand: number of features in random segments, #Rand: number of random segments, %inRand: percent of features in random segments, LogOR: log-odd-ratio of the logistic regression to test enrichment in Oris with respect to enrichment in random segments (see Material & Methods). When considering origins with pG4s, we restrict the analysis to segments that carry a pG4 (Oris and Random). By doing so we test whether, when origins or random segments are associated with a pG4, the enrichment concerns the monomeric or the Clustered form.

Strong Oris Human H9 SNS (38,849 Strong Oris)							
All Oris	#inOri	#Ori	%inOri	#inRand	#Rand	%inRand	LogOR
CGI	24,786	77,698	32	4,462	72,548	6	2***
TSS	12,147	77,698	16	2,056	72,548	3	1.8***
all pG4s	60,260	77,698	78	23,554	72,548	32	2***
Monomeric pG4s	48,374	77,698	62	20,940	72,548	29	1.4***
Clustered pG4s	29,234	77,698	38	4,499	72,548	6	2.2***
H2AZ	20,614	77,698	26	9,924	72,548	14	0.7***
chIP G4, Zheng et al. 2020	67,232	77,698	86	26,380	72,548	36	2.4***
chIP G4, Hansel-Hertsch et al. 2016	4,009	77,698	5	997	72,314	1	1.4***
Oris with pG4	#inOri	#Ori	%inOri	#inRand	#Rand	%inRand	LogOR
CGI	20,600	60,260	34	2,625	23,554	11	1.4***
TSS	10,480	60,260	17	1,179	23,554	5	1.4***
Monomeric pG4s	48,374	60,260	80	20,940	23,554	89	-0.7***
Clustered pG4s	29,234	60,260	48	4,499	23,554	19	1.4***
H2AZ	16,569	60,260	28	3,520	23,554	15	0.8***
chIP G4, Zheng et al. 2020	53,823	60,260	89	12,974	23,554	55	1.9***
chIP G4, Hansel-Hertsch et al. 2016	3,339	60,260	6	493	23,177	2	1***

Strong Oris Human EJ30 from Guilbaud et al. (2022), Iniseq2 (5,953 Strong Oris)							
All Oris	#inOri	#Ori	%inOri	#inRand	#Rand	%inRand	LogOR
CGI	11,494	11,906	96	121	11,908	1	7.9***
TSS	8,886	11,906	75	85	11,908	1	6***
all pG4s	9,673	11,906	81	1,821	11,908	15	3.2***
Monomeric pG4s	7,644	11,906	64	1,682	11,908	14	2.4***
Clustered pG4s	4,626	11,906	39	190	11,908	2	3.7***
chIP G4, Zheng et al. 2020	11,719	11,906	98	1,530	11,908	13	6.1***
chIP G4, Hansel-Hertsch et al. 2016	3,218	11,906	27	68	11,908	1	4.2***
Oris with pG4	#inOri	#Ori	%inOri	#inRand	#Rand	%inRand	LogOR
CGI	9,384	9,673	97	37	1,821	2	7.4***
TSS	7,234	9,673	75	20	1,821	1	5.6***
Monomeric pG4s	7,644	9,673	79	1,682	1,821	92	-1.2***
Clustered pG4s	4,626	9,673	48	190	1,821	10	2.1***
chIP G4, Zheng et al. 2020	9,543	9,673	99	462	1,821	25	5.4***
chIP G4, Hansel-Hertsch et al. 2016	2,551	9,673	26	16	1,838	1	3.7***

Table 5: Enrichment of Strong Origins in genomic features in human cells. Strong origins: top 25% most active origins in terms of SNS enrichment. Associations were computed separately 1kb downstream (pG4 on the minus strand) and 1 kb upstream the ori peak (pG4 on the plus strand), then combined so the total number of origins corresponds to 2 times the initial number indicated in the table's header. Strong Oris with pG4s consist in strong origins that carry a pG4. #inOri: number of features in Strong Oris, #Ori: number of Strong Oris, %inOri: percent of features in Strong Oris, #inRand: number of features in random segments, #Rand: number of random segments, %inRand: percent of features in random segments, LogOR: log-odd-ratio of the logistic regression to test enrichment in Strong Oris with respect to enrichment in random segments (see Material & Methods). When considering strong origins with pG4s, we restrict the analysis to segments that carry a pG4 (Strong Oris and Random). By doing so we test whether, when strong origins or random segments are associated with a pG4, the enrichment concerns the monomeric or the Clustered form.

Strong Oris Chicken DT40, SNS (17,017 Strong Oris)							
All Oris	#inOri	#Ori	%inOri	#inRand	#Rand	%inRand	LogOR
CGI	16,085	34,034	47	3,995	31,589	13	1.8***
TSS	3,482	34,034	10	566	31,589	2	1.8***
all pG4s	25,746	34,034	76	9,895	31,589	31	1.9***
Monomeric pG4s	20,909	34,034	61	8,866	31,589	28	1.4***
Clustered pG4s	12,232	34,034	36	2,064	31,652	6	2.1***
H2AZ	24,968	34,034	73	17,302	31,589	55	0.8***
NFR in G1	5,864	34,034	17	1,755	31,589	6	1.3***
NFR in G2	4,953	34,034	15	1,157	31,589	4	1.5***
chIP G4, Zheng et al. 2020	9,576	34,034	28	2,487	31,589	8	1.5***
Oris with pG4	#inOri	#Ori	%inOri	#inRand	#Rand	%inRand	LogOR
CGI	13,127	25,746	51	2,511	9,895	25	1.1***
TSS	2,959	25,746	12	403	9,895	4	1.1***
Monomeric pG4s	20,909	25,746	81	8,866	9,895	90	-0.7***
Clustered pG4s	12,232	25,746	48	1,999	9,895	20	1.3***
H2AZ	19,381	25,746	75	6,488	9,895	66	0.5***
NFR in G1	4,668	25,746	18	861	9,895	9	0.8***
NFR in G2	4,063	25,746	16	589	9,895	6	1.1***
chIP G4, Zheng et al. 2020	7,664	25,746	30	1,248	9,895	13	1.1***
**Strong Oris Mouse mESC, SNS (51,348 Strong Oris)**							
All Oris	#inOri	#Ori	%inOri	#inRand	#Rand	%inRand	LogOR
CGI	26,816	102,696	26	627	47,614	1	3.3***
TSS	21,519	102,696	21	888	47,614	2	2.6***
all pG4s	60,685	102,696	59	13,903	47,614	29	1.3***
Monomeric pG4s	50,942	102,696	50	12,438	47,614	26	1***
Clustered pG4s	18,605	102,696	18	1,986	47,614	4	1.6***
H2AZ	38,409	102,696	37	5,140	47,614	11	1.6***
chIP G4, Zheng et al. 2020	28,935	102,696	28	1,901	47,614	4	2.2***
Oris with pG4	#inOri	#Ori	%inOri	#inRand	#Rand	%inRand	LogOR
CGI	18,529	60,685	30	299	13,903	2	3***
TSS	15,300	60,685	25	383	13,903	3	2.5***
Monomeric pG4s	50,942	60,685	84	12,438	13,903	90	-0.5***
Clustered pG4s	18,605	60,685	31	1,986	13,903	14	1***
H2AZ	25,730	60,685	42	2,006	13,903	14	1.5***
chIP G4, Zheng et al. 2020	20,124	60,685	33	830	13,903	6	2.1***

Table 6: Enrichment of Strong Origins in genomic features in chicken and mouse cells. Strong origins: top 25% most active origins in terms of SNS enrichment. Associations were computed separately 1kb downstream (pG4 on the minus strand) and 1 kb upstream the ori peak (pG4 on the plus strand), then combined so the total number of origins corresponds to 2 times the initial number indicated in the table's header. Strong Oris with pG4s consist in strong origins that carry a pG4. #inOri: number of features in Strong Oris, #Ori: number of Strong Oris, %inOri: percent of features in Strong Oris, #inRand: number of features in random segments, #Rand: number of random segments, %inRand: percent of features in random segments, LogOR: log-odd-ratio of the logistic regression to test enrichment in Strong Oris with respect to enrichment in random segments (see Material & Methods). When considering strong origins with pG4s, we restrict the analysis to segments that carry a pG4 (Strong Oris and Random). By doing so we test whether, when strong origins or random segments are associated with a pG4, the enrichment concerns the monomeric or the Clustered form.

H2AZ Human H9 (220,029 peaks)							
All H2AZ	#inH2AZ	#H2AZ	%inH2AZ	#inRand	#Rand	%inRand	LogOR
CGI	48,517	440,058	11	8,140	706,920	1	2.4***
TSS	35,253	440,058	8	4,309	706,920	1	2.7***
Oris	81,497	440,058	18	52,589	706,920	7	1***
Strong Oris	26,295	440,058	6	12,304	706,920	2	1.3***
Monomeric pG4s	92,474	440,058	21	94,468	706,920	13	0.5***
Clustered pG4	22,924	440,058	5	15,414	706,920	2	0.9***
chIP G4, Zheng et al. 2020	120,845	440,058	28	92,154	706,920	13	0.9***
chIP G4, Hansel-Hertsch et al. 2016	17,006	440,058	4	2,074	706,920	0	2.6***
H2AZ with pG4	#inH2AZ	#H2AZ	%inH2AZ	#inRand	#Rand	%inRand	LogOR
CGI	31,486	104,376	30	5,081	104,032	5	2.1***
TSS	22,261	104,376	21	1,827	104,032	2	2.7***
Oris	43,335	104,376	42	23,101	104,032	22	0.9***
Strong Oris	19,378	104,376	19	8,383	104,032	8	1***
Monomeric pG4s	92,474	104,376	89	94,468	104,032	91	-0.2***
Clustered pG4	22,924	104,376	22	15,414	104,032	15	0.5***
chIP G4, Zheng et al. 2020	59,400	104,376	57	35,933	104,032	34	0.9***
chIP G4, Hansel-Hertsch et al. 2016	9,458	104,376	9	741	104,032	1	2.6***

H2AZ Chicken DT40 (269,829 peaks)							
All H2AZ	#inH2AZ	#H2AZ	%inH2AZ	#inRand	#Rand	%inRand	LogOR
CGI	55,537	539,658	10	9,087	588,604	2	2***
TSS	14,730	539,658	3	1,313	588,604	0	2.5***
Oris	114,184	539,658	21	42,588	588,604	7	1.2***
Strong Oris	35,820	539,658	7	7,557	588,604	1	1.7***
Monomeric pG4s	117,530	539,658	22	48,331	588,604	8	1.1***
Clustered pG4	30,601	539,658	6	6,089	588,604	1	1.7***
NFR in G1	41,200	539,658	8	11,067	588,604	2	1.5***
chIP G4, Zheng et al. 2020	51,859	539,658	10	13,286	588,604	2	1.5***
H2AZ with pG4	#inH2AZ	#H2AZ	%inH2AZ	#inRand	#Rand	%inRand	LogOR
CGI	35,204	133,606	26	4,753	52,195	9	1.3***
TSS	9,622	133,606	7	526	52,195	1	2***
Oris	50,028	133,606	37	10,148	52,195	19	0.9***
Strong Oris	24,724	133,606	18	4,329	52,195	8	0.9***
Monomeric pG4s	117,530	133,606	88	48,331	52,195	93	-0.5***
Clustered pG4	30,601	133,606	23	6,089	52,195	12	0.8***
NFR in G1	16,356	133,606	12	1,545	52,195	3	1.5***
chIP G4, Zheng et al. 2020	25,257	133,606	19	2,872	52,195	6	1.4***

H2AZ Mouse mESC (78,359 peaks)							
All H2AZ	#inH2AZ	#H2AZ	%inH2AZ	#inRand	#Rand	%inRand	LogOR
CGI	31,504	156,718	20	2,006	150,554	1	2.9***
TSS	28,834	156,718	18	2,588	150,554	2	2.6***
Oris	80,498	156,718	51	34,420	150,554	23	1.3***
Strong Oris	42,588	156,718	27	10,959	150,554	7	1.6***
Monomeric pG4s	61,985	156,718	40	38,099	150,554	25	0.7***
Clustered pG4	16,025	156,718	10	7,082	150,554	5	0.8***
chIP G4, Zheng et al. 2020	36,139	156,718	23	5,304	150,554	4	2.1***
H2AZ with pG4	#inH2AZ	#H2AZ	%inH2AZ	#inRand	#Rand	%inRand	LogOR
CGI	19,924	70,898	28	1,026	43,146	2	2.8***
TSS	17,618	70,898	25	1,215	43,146	3	2.4***
Oris	43,058	70,898	61	13,663	43,146	32	1.2***
Strong Oris	26,635	70,898	38	5,448	43,146	13	1.4***
Monomeric pG4s	61,985	70,898	87	38,099	43,146	88	-0.1***
Clustered pG4	16,025	70,898	23	7,082	43,146	16	0.4***
chIP G4, Zheng et al. 2020	21,675	70,898	31	2,549	43,146	6	1.9***

Table 7: Enrichment of H2AZ peaks in genomic features. Associations were computed separately 1kb downstream (pG4 on the minus strand) and 1 kb upstream the H2AZ peak (pG4 on the plus strand), then combined so the total number of peaks corresponds to 2 times the initial number indicated in the table's header. H2AZ with pG4s consist in H2AZ that carry a pG4. #inH2AZ: number of features in H2AZ, #H2AZ: number of H2AZ, %inH2AZ: percent of features in H2AZ, #inRand: number of features in random segments, #Rand: number of random segments, %inRand: percent of features in random segments, LogOR: log-odd-ratio of the logistic regression to test enrichment in H2AZ with respect to enrichment in random segments (see Material & Methods). When considering H2AZ with pG4s, we restrict the analysis to segments that carry a pG4 (H2AZ and Random). By doing so we test whether, when H2AZ or random segments are associated with a pG4, the enrichment concerns the monomeric or the clustered form.

NFR Chicken DT40 (23,128 NFRs)							
All NFR	#inNFR	#NFR	%inNFR	#inRand	#Rand	%inRand	LogOR
CGI	15,808	46,256	34	7,163	46,256	16	1***
TSS	7,581	46,256	16	943	46,256	2	2.2***
Oris	17,015	46,256	37	10,492	46,256	23	0.7***
Strong Oris	7,274	46,256	16	5,550	46,256	12	0.3***
All pG4s	16,803	46,256	36	15,158	46,256	33	0.2***
Monomeric pG4s	13,918	46,256	30	13,136	46,256	28	0.1***
Clustered pG4s	6,157	46,256	13	4,310	46,256	9	0.4***
H2AZ	33,954	46,256	73	23,327	46,256	50	1***
NFR with pG4	#inNFR	#NFR	%inNFR	#inRand	#Rand	%inRand	LogOR
CGI	10,259	16,803	61	4,920	15,158	32	1.2***
TSS	5,119	16,803	30	658	15,158	4	2.3***
Oris	9,932	16,803	59	5,976	15,158	39	0.8***
Strong Oris	5,218	16,803	31	4,217	15,158	28	0.2***
Clustered pG4s	6,157	16,803	37	4,310	15,158	28	0.4***
Monomeric pG4s	13,918	16,803	83	13,136	15,158	87	-0.3***
H2AZ	14,961	16,803	89	10,045	15,158	66	1.4***

Table 8: Enrichment of NFR (in G1) in genomic features. Associations were computed separately 1kb downstream (pG4 on the minus strand) and 1 kb upstream the NFR (pG4 on the plus strand), then combined so the total number of NFR corresponds to 2 times the initial number indicated in the table's header. NFR with pG4s consist in NFRs that carry a pG4. #inNFR: number of features in NFR, #NFR: number of NFRs, %inNFR: percent of features in NFR, #inRand: number of features in random segments, #Rand: number of random segments, %inRand: percent of features in random segments, LogOR: log-odd-ratio of the logistic regression to test enrichment in NFRs with respect to enrichment in random segments (see Material & Methods). When considering NFRs with pG4s, we restrict the analysis to segments that carry a pG4 (NFRs and Random). By doing so we test whether, when NFRs or random segments are associated with a pG4, the enrichment concerns the monomeric or the clustered form.

	Forward primer sequence	Reverse primer sequence	Genomic position (Build Mars 2018)
PBNs enrichment analysis			
Primers 0	GGGCTATTCAGCTTGTCTAG	GCCACCTCAACTTTTGTATAC	
Primers 1	GGGGACTGCTCACGTTCATCA	AATGTGGCGTGTGGGATCTC	
Primers 1'	CTACACAGAGGTCCTGCTG	GTGAAGAGAAGCCTCAGGCA	
Primers 2	GGGAGCAAGAGCCCAGAC	GTGAGCAGTCCCCACATCAG	
Primers 3	GGGCTATTCAGCTTGTCTAG	TGGAACGTAACTGCGCAACT	
Primers 4	GGGCTATTCAGCTTGTCTAG	GTATACAAAGTTGCTCTTGTG	
Bkgd	TCCATACAGCCACAACAGCA	TGTGGAAGAGTTTCAGTCCAGG	chr1:72804257+72804372
ρ-globin	GACGGTCAGGTTTGCCAAAG	TCCTGAGGATACGTTTTTCAG	chr1:197287850-197288114
Replication timing analysis			
Early	GACGGTCAGGTTTGCCAAAG	TCCTGAGGATACGTTTTTCAG	chr1:197287850-197288114
With	GGGGACTGCTCACGTTCATCA	AATGTGGCGTGTGGGATCTC	
Both	TCCATACAGCCACAACAGCA	TGTGGAAGAGTTTCAGTCCAGG	chr1:72804257+72804372
Without	CAGGACAGCAGGTATTCACA	GGCCTGAACACTGTGTCAAT	chr1:72798802+72798956
Screening of targeted integration			
5'-screening-site (primers 1 and 2)	GTGCAGCATCAGTGGATAAAGT	GCCACCTCAACTTTTGTATAC	
5'-screening-site (primers 1 and 2')	GTGCAGCATCAGTGGATAAAGT	TGGAACGTAACTGCGCAACT	
3'-screening-site (primers 5a and 6) allele 1	CTACACAGAGGTCCTGCTG	CCACATGTTTATTGCATACGGC	
3'-screening-site (primers 5b and 6) allele 2	CGGCAGTACATATTGAAGCGT	CCACATGTTTATTGCATACGGC	
Screening of site specific excision			
BsR cassette excision (primers 3 and 4)	CCCCCTGAACCTGAAACATAA	CCAGGCTGTACTCTGAATCATCT	
BsR cassette excision (primers 3' and 4)	TGTATACAAAAGTTGCGCAGTTACGTTCCA	CCAGGCTGTACTCTGAATCATCT	
Copy number quantification			
With (β^A-globin constructs)	GGGGACTGCTCACGTTCATCA	AATGTGGCGTGTGGGATCTC	
With (Med14 constructs)	GGGCTATTCAGCTTGTCTAG	TGGAACGTAACTGCGCAACT	
Both	TCCATACAGCCACAACAGCA	TGTGGAAGAGTTTCAGTCCAGG	chr1:72804257+72804372
RNA quantification			
Med14 gene	TGGGCTAATAATGCTGGAAAGGT	TAGAGAAGCCAGACGATCAGCA	chr1:113541601+113542342
Il2R gene	GGGGACTGCTCACGTTCATCA	AATGTGGCGTGTGGGATCTC	
Primers 1 and 2	GGGCTATTCAGCTTGTCTAG	AATGTGGCGTGTGGGATCTC	

Table 9: Primer sets used for quantitative PCR

	With concentration	Both concentration	Ratio With/Both
ΔCAAT #1	3.49	6.79	0.51
ΔCAAT #2	3.51	7.01	0.50
ΔTATA #1	4.04	9.02	0.45
ΔTATA #2	3.83	7.75	0.49
ΔTATA #3	7.98	18.50	0.43
ΔTATA #4	6.28	14.57	0.43
ΔTATA #5	4.86	11.30	0.43
ΔCAAT+ΔTATA #1	0.49	0.97	0.50
ΔCAAT+ΔTATA #2	3.68	7.95	0.46
ΔCACC #1	3.61	7.25	0.50
ΔCACC #2	3.75	7.22	0.52
ΔCACC #3	6.13	13.60	0.45
Δ(CAAT to TATA) #1	3.33	7.02	0.48
Δ(CAAT to TATA) #2	3.36	6.91	0.49
ΔCAAT+ΔTATA mpG4 #1	0.31	0.57	0.55
ΔCAAT+ΔTATA mpG4 #2	0.44	0.75	0.58
β^A -globin minimal origin #1	7.15	15.00	0.48
β^A -globin minimal origin #2	0.76	1.17	0.65
β^A -globin minimal origin #3	0.40	0.75	0.54
β^A -globin minimal origin #4	6.80	15.27	0.45
β^A -globin minimal origin #5	6.25	13.97	0.45
β^A -globin minimal origin pG4#1 rev compl #1	4.02	6.72	0.60
β^A -globin minimal origin pG4#1 rev compl #2	0.49	0.97	0.50
2x β^A-globin minimal origin #1	0.51	0.63	0.81
2x β^A -globin minimal origin pG4#1 rev compl #1	0.35	0.37	0.92
ΔpG4#1 pG4#2 Compl #1	0.32	0.56	0.57
ΔpG4#1 pG4#2 Compl #2	6.60	14.87	0.44
ΔpG4#1 pG4#2 Compl #3	0.30	0.53	0.56
ΔpG4#1 pG4#2 Compl #4	5.50	12.80	0.43
β^A -globin minimal origin ΔCAAT+ ΔTATA #1	0.57	1.09	0.52
βA -globin minimal origin ΔCAAT+ ΔTATA #2	0.50	0.91	0.55
β^A -globin minimal origin ΔCAAT+ ΔTATA #3	0.65	1.38	0.47
Med14 pG4#4+5 origin #1	0.51	1.08	0.47
Med14 pG4#4+5 origin #2	0.52	0.99	0.53
Med14 pG4#4+5 minimal origin #1	0.52	1.15	0.46
Med14 pG4#4+5 minimal origin #2	0.56	1.14	0.49

Table 10: Transgene copy number determination in clonal cell lines.
The table shows the qPCR results obtained with genomic DNA extracted from the clones selected for the experiments. For each clone, 2 ng of genomic DNA was amplified with a primer set amplifying a sequence within the construct (With) and another primer set amplifying a sequence 5 kb downstream from the insertion site for both alleles (Both). The ratio of the amounts of DNA obtained with the With and Both primer sets was used to determine transgene copy number in all clonal cell lines.

Supplementary Figures Source Data

Supplementary Figure 1c

M (lane 9) / β^{A}-globin full origin cDNA (lane 10-11) / β^{A}-globin full origin gDNA (lane 12)

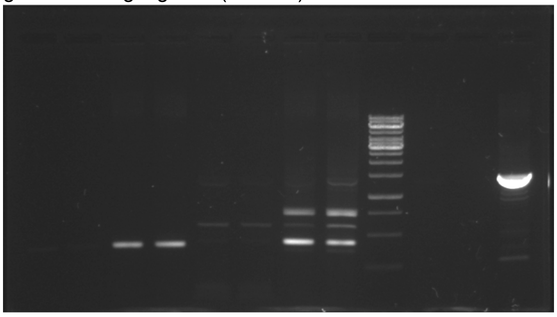

M (lane 2) / β^{A}-globin minimal origin cDNA (lane 3-4) / β^{A}-globin minimal origin gDNA (lane 5) / M (lane 6)

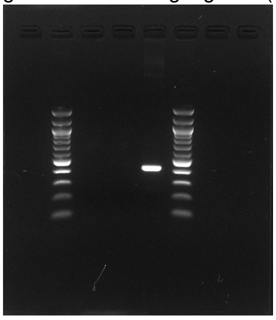

Supplementary Figure 14b

β^{A}-globin minimal origin (lanes 1-5) / β^{A}-globin minimal origin pG4#1 rev compl (lanes 6-7) / M (lane 8) / ΔCACC (lanes 9-11)/ ΔCAAT (lanes 12-13) / Δ(CAAT to TATA) (lanes 14-15) / M (lane 16) / Wt (lane 17)

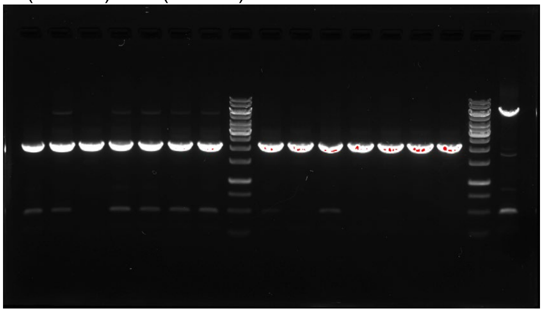

M (lane 1) / β^{A}-globin minimal origin ΔCAAT+ ΔTATA (lanes 2-4) / M (lane 5) / Wt (lane 6)

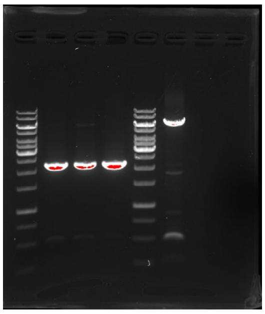

ΔCAAT+ ΔTATA (lane 1-2) / ΔTATA (lane 3-7) / M (lane 8)/ β^{A}-globin ΔpG4#1 pG4#2 Compl (lane 9-12) / ΔCAAT+ ΔTATA mpG4 (lane 13-14) / M (lane 15) / Wt (lane 16)

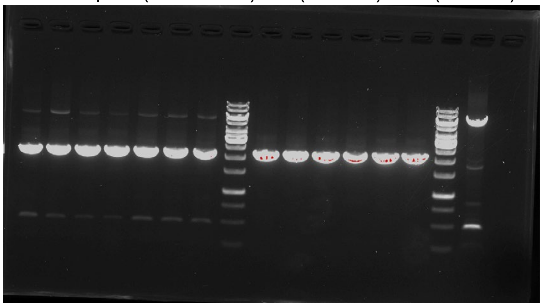

Med14 pG4#4+5 origin (lane 2-3) / M (lane 4)/ Med14 pG4#4+5 minimal origin (lane 5-6)

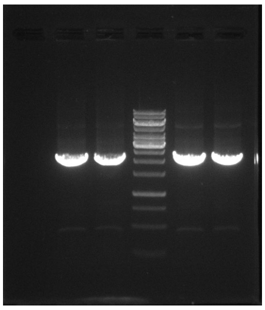

Supplementary Figure 14c

ΔCACC (lane 1-3) / ΔCAAT (lane 4-5) / Δ(CAAT to TATA) (lane 6-7) / M (lane 8) / ΔCAAT+ ΔTATA (lanes 9-10) / ΔTATA (lane 11-15) / M (lane 16) / Wt (lane 17)

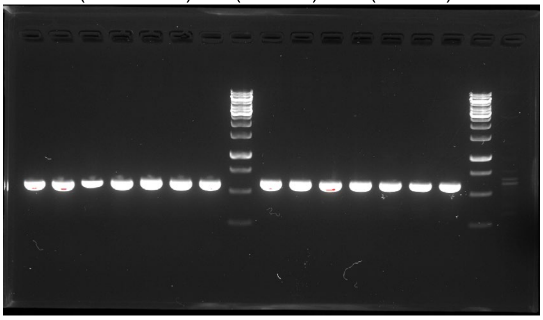

M (lane 1) / β^A-globin minimal origin ΔCAAT+ ΔTATA (lanes 2-4) / M (lane 5) / Wt (lane 6)

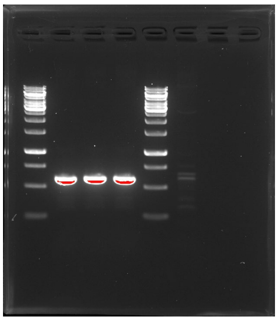

ΔCAAT+ ΔTATA mpG4 (lane 1-2) / β^A-globin ΔpG4#1 pG4#2 Compl (lane 3-6) / β^A-globin minimal origin (lane 7-11) / β^A-globin minimal origin pG4#1 rev compl (lanes 12-13) / M (lane 14) / Wt (lane 15)

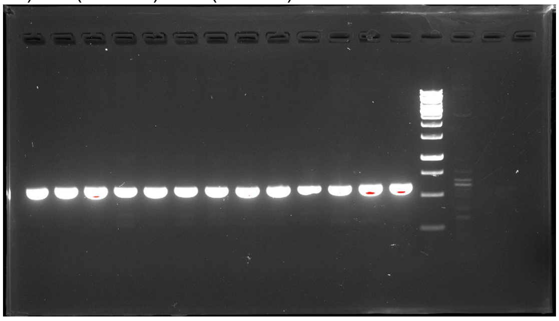

Med14 pG4#4+5 origin (lane 2-3) / M (lane 4) / Med14 pG4#4+5 minimal origin (lane 5-6)

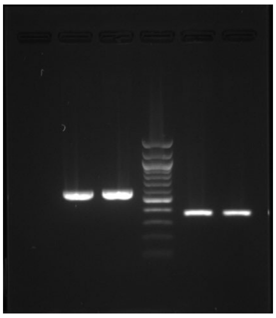

Supplementary Figure 14e

2xβ^A-globin minimal origin (lane 1) / 2xβ^A-globin minimal origin pG4#1 rev compl (lane 2) / Wt (lane 3) / M (lane 4) / 2xβ^A-globin minimal origin (lane 5) / 2xβ^A-globin minimal origin pG4#1 rev compl (lane 6) / Wt (lane 7) / M (lane 8)

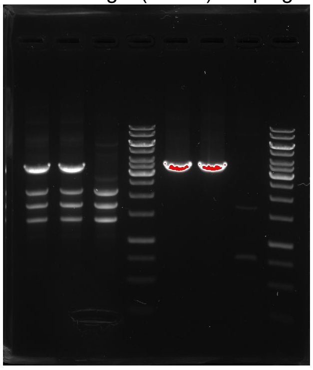

Supplementary Figure 14f

M (lane 1) / 2xβ^A-globin minimal origin (lane 2) / 2xβ^A-globin minimal origin pG4#1 rev compl (lane 3) / M (lane 4)) / Wt (lane 5)

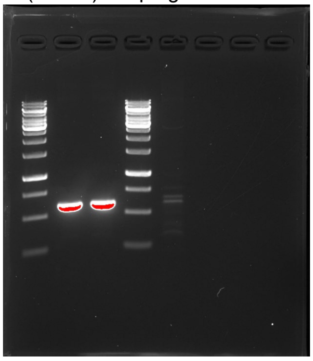

Supplementary Figure 15a

M (lane 1) / β^A-globin minimal origin : G1/S transition synchronized cells (lanes 2-5; 2,5U-10U-40U-160U) / G2 transition synchronized cells (lanes 6-9; 2,5U-10U-40U-160U)

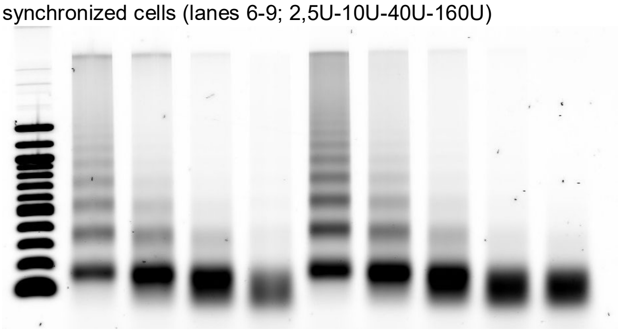

Supplementary Figure 15b

M (lane 1) / β^A-globin minimal origin pG4#1 rev compl : G1/S transition synchronized cells (lanes 2-5; 2,5U-10U-40U-160U) / G2 transition synchronized cells (lanes 6-9; 2,5U-10U-40U-160U)

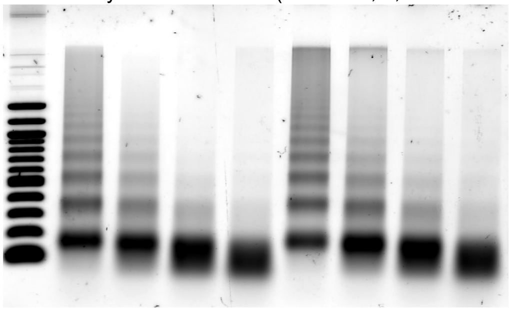

Supplementary Figure 15c

M (lane 1) / β^A-globin minimal origin Wt 160U (lane 2) / β^A-globin minimal origin pG4#1 rev compl 160U (lane 3) / M (lane 4)

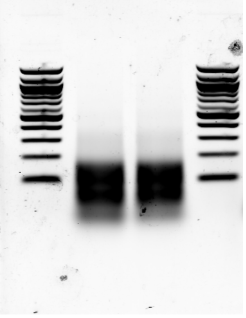

Supplement: Supplementary file 1 — Supplementary information [file 41467_2023_40441_MOESM1_ESM.pdf]
