# Supplementary material for: Dimeric G-quadruplex motifs-induced NFRs determine strong replication origins in vertebrates
Source: Nat Commun. 2023 Aug 10;14:4843. doi: 10.1038/s41467-023-40441-4 (PMC10415359; doi:10.1038/s41467-023-40441-4)
Supplement: Supplementary file 3 — Reporting Summary [file 41467_2023_40441_MOESM3_ESM.pdf]

nature portfolio

Corresponding author(s): Marie-Noëlle Prioleau
Franck Picard

Last updated by author(s): Jul 19, 2023

Reporting Summary

Nature Portfolio wishes to improve the reproducibility of the work that we publish. This form provides structure for consistency and transparency in reporting. For further information on Nature Portfolio policies, see our Editorial Policies and the Editorial Policy Checklist.

Statistics

For all statistical analyses, confirm that the following items are present in the figure legend, table legend, main text, or Methods section.

n/a	Confirmed	
☐	☒	The exact sample size (*n*) for each experimental group/condition, given as a discrete number and unit of measurement
☐	☒	A statement on whether measurements were taken from distinct samples or whether the same sample was measured repeatedly
☐	☒	The statistical test(s) used AND whether they are one- or two-sided<br>*Only common tests should be described solely by name; describe more complex techniques in the Methods section.*
☐	☒	A description of all covariates tested
☒	☐	A description of any assumptions or corrections, such as tests of normality and adjustment for multiple comparisons
☐	☒	A full description of the statistical parameters including central tendency (e.g. means) or other basic estimates (e.g. regression coefficient) AND variation (e.g. standard deviation) or associated estimates of uncertainty (e.g. confidence intervals)
☐	☒	For null hypothesis testing, the test statistic (e.g. *F*, *t*, *r*) with confidence intervals, effect sizes, degrees of freedom and *P* value noted<br>*Give P values as exact values whenever suitable.*
☒	☐	For Bayesian analysis, information on the choice of priors and Markov chain Monte Carlo settings
☒	☐	For hierarchical and complex designs, identification of the appropriate level for tests and full reporting of outcomes
☒	☐	Estimates of effect sizes (e.g. Cohen's *d*, Pearson's *r*), indicating how they were calculated

Our web collection on statistics for biologists contains articles on many of the points above.

Software and code

Policy information about availability of computer code

Data collection	The mapping to the chicken gal5 genome using version 2.3.4.1 of bowtie2. Duplicated reads were removed using Picard Toolkit v2. 20 (http://broadinstitute.github.io/picard/). The BD FACSDiva software v 8.0.1 was used for flow cytometry.
Data analysis	Overlaps between genomic features were performed using intersectBed of the BedTools. Random segments were sampled using the random function of BedTools (Quinlan, A. R et al. 2010). The significance of enrichments was assessed using logistic regression (Faraway, J. J et al. 2016).<br>For ATAC-seq datas, we called open regions using MAC2s version 2.1.2. NFRs were called in open regions using the nucleoatac pipeline (Schep, A. N et al. 2015).<br>H2AZ peak detection was performed using MAC2s version 2.1.2.<br>Nucleosome occupancy profiles shown in Figure 4 were generated using the bamCoverage tool on the Galaxy platform (v3.5.1) with the following parameters: Bin size in bases =10; scaling/normalization method: 1X and in advanced options: smooth values=50; Minimum fragment length =100; Maximum=500; determine nucleosome positions form Mnase-seq= True.<br>For RT analysis, centered and standardized profiles were smoothed using cubic splines (smooth.spline function of R).

For manuscripts utilizing custom algorithms or software that are central to the research but not yet described in published literature, software must be made available to editors and reviewers. We strongly encourage code deposition in a community repository (e.g. GitHub). See the Nature Portfolio guidelines for submitting code & software for further information.

Data

Policy information about availability of data

All manuscripts must include a data availability statement. This statement should provide the following information, where applicable:

- Accession codes, unique identifiers, or web links for publicly available datasets
- A description of any restrictions on data availability
- For clinical datasets or third party data, please ensure that the statement adheres to our policy

The genome-wide data used in the current study have been either generated for the purpose of the study or download from database as described in the methods section. All sequencing files and processed bed files were deposited in Gene Omnibus (GEO) with accession number GSE231492. Data have been released public on July 19, 2023.

Human research participants

Policy information about studies involving human research participants and Sex and Gender in Research.

Reporting on sex and gender	not applicable
Population characteristics	not applicable
Recruitment	not applicable
Ethics oversight	not applicable

Note that full information on the approval of the study protocol must also be provided in the manuscript.

Field-specific reporting

Please select the one below that is the best fit for your research. If you are not sure, read the appropriate sections before making your selection.

☒ Life sciences ☐ Behavioural & social sciences ☐ Ecological, evolutionary & environmental sciences

For a reference copy of the document with all sections, see nature.com/documents/nr-reporting-summary-flat.pdf

Life sciences study design

All studies must disclose on these points even when the disclosure is negative.

Sample size	For each Replication timing or SNS enrichment analysis at least two independant clones were analyzed. Sample sizes were chosen as a compromise between the amount of work and the significance of the results, based on our years-long experience with the technique used (Valton, A.-L. et al. G4 motifs affect origin positioning and efficiency in two vertebrate replicators. EMBO J. 33, 732–746 (2014)). All sample size are indicated in the figures.
Data exclusions	No data were excluded from the reported analyses
Replication	Each quantification was performed at least with duplicates the standard deviation was calculated. For replicated experiments, all attempts at replication were successful.
Randomization	Most of the time, samples from one or two clones were processed in parallel for genetic analyses. Randomization is not relevant to our study. For sequencing experiments, samples were randomly assigned to the flow cells.
Blinding	For each experiment we used at least two different clones that were randomly selected after drug resistance selection. Investigators were not blinded to group allocation. There is no rationale as to why blinding was not performer. This is just the way the experiments were performed.

Reporting for specific materials, systems and methods

We require information from authors about some types of materials, experimental systems and methods used in many studies. Here, indicate whether each material, system or method listed is relevant to your study. If you are not sure if a list item applies to your research, read the appropriate section before selecting a response.

Materials & experimental systems

n/a	Involved in the study
☐	☒ Antibodies
☐	☒ Eukaryotic cell lines
☒	☐ Palaeontology and archaeology
☒	☐ Animals and other organisms
☒	☐ Clinical data
☒	☐ Dual use research of concern

Methods

n/a	Involved in the study
☐	☒ ChIP-seq
☐	☒ Flow cytometry
☒	☐ MRI-based neuroimaging

Antibodies

Antibodies used

anti-H2A.Z antibody (Active Motif, #39013); anti-BrdU antibody (BD Biosciences #347580)

Validation

The anti-H2A.Z antibody from active motif has been validated for use in ChIP and ChIP-Seq in human. H2A.Z is highly conserved during evolution which led us to believe that it will be effective in our avian lineage. As a positive control for immunoprecipitation, we quantified the H2A.Z enrichment by qPCR on a poised nucleosome located upstream of the NFR of the Med14 gene (active in DT40). Each immunoprecipitation was made in a final volume of 300µl with the amount of MNAse digested chromatin corresponding to 10µg of DNA and 10µl of anti-H2A.Z antibody.

We used the anti-BrdU antibody from BD Biosciences for several years for replication timing analysis in DT40 cell lines (Hassan-Zadeh 2012, Valton 2014, Brossas 2020). It as purified from the clone B44, derived from hybridization of mouse Sp2/0-Ag14 myeloma cells with spleen cells from BALB/c mice immunized with iodouridine-conjugated ovalbumin.
The anti-BrdU antibody is supplied at 25µg/mL. We used 40µl (=1µg) per immunoprecipitation of 5x10^4 exponentially growing cells labelled for one hour with 50µM BrdU.

Eukaryotic cell lines

Policy information about cell lines and Sex and Gender in Research

Cell line source(s)

DT40Cre1 cell line has been described previously (Arakawa et al. 2001). This cell line was obtained from the Arakawa lab as a kind gift.

Authentication

The karyotype of the wild type DT40 cell line was verified and displayed a typical DT40 karyotype nearly diploid with a trisomy of chromosome 2 in Hassan-Zadeh et al. 2012.

Mycoplasma contamination

The DT40 Wt cell line was tested for mycoplasma contamination upon reception from the H. Arakawa's lab and then several clones obtained were tested repeatedly. We have never detected mycoplasma contamination in either our Wt or mutant lines.

Commonly misidentified lines
(See ICLAC register)

none

ChIP-seq

Data deposition

☒ Confirm that both raw and final processed data have been deposited in a public database such as GEO.

☒ Confirm that you have deposited or provided access to graph files (e.g. BED files) for the called peaks.

Data access links
May remain private before publication.

To review GEO accession GSE231492:
Go to https://www.ncbi.nlm.nih.gov/geo/query/acc.cgi?acc=GSE231492

Files in database submission

ChIP-seq files: Raw sequencing data file
chipfiles :

ChIp_H2AZ_2xMinikirikou_12-7_S3_L001_R1_001.fastq.gz
ChIp_H2AZ_2xMinikirikou_12-7_S3_L001_R2_001.fastq.gz
ChIp_H2AZ_2xMinikirikou_12-7_S3_L002_R1_001.fastq.gz
ChIp_H2AZ_2xMinikirikou_12-7_S3_L002_R2_001.fastq.gz
ChIp_H2AZ_2xMinikirikou_12-7_S3_L003_R1_001.fastq.gz
ChIp_H2AZ_2xMinikirikou_12-7_S3_L003_R2_001.fastq.gz
ChIp_H2AZ_2xMinikirikou_12-7_S3_L004_R1_001.fastq.gz
ChIp_H2AZ_2xMinikirikou_12-7_S3_L004_R2_001.fastq.gz

control files :

Input_2xMinikirikou_12-7_S1_L001_R1_001.fastq.gz

Input_2xMinikirikou_12-7_S1_L001_R2_001.fastq.gz
Input_2xMinikirikou_12-7_S1_L002_R1_001.fastq.gz
Input_2xMinikirikou_12-7_S1_L002_R2_001.fastq.gz
Input_2xMinikirikou_12-7_S1_L003_R1_001.fastq.gz
Input_2xMinikirikou_12-7_S1_L003_R2_001.fastq.gz
Input_2xMinikirikou_12-7_S1_L004_R1_001.fastq.gz
Input_2xMinikirikou_12-7_S1_L004_R2_001.fastq.gz

index file : galGal5.fa

Genome browser session (e.g. UCSC): no longer applicable

Methodology

Replicates: Two ChIP-seq experiments were performed and validated by qPCR quantification at specific genomic positions.

Sequencing depth: For H2A.Z ChIp analyses, 200M of paired-end reads were generated. The sequencing size is 75 bp.

Antibodies: anti-H2A.Z antibody (Active Motif, #39013; lot N07020001)

Peak calling parameters:
H2AZ peak detection was performed using the following parameters: macs 2.1.2 --macs_gsize 1.2e9
effective genome size = 1.20e+09
band width = 300
model fold = [5, 50]
qvalue cutoff = 5.00e-02

Data quality: nb of peaks at FDR 5% and above 5-fold enrichment: 285,028, Above 5 fold : 16526

Software:
command line program:

bowtie2 2.3.4.1 https://doi.org/10.1038/nmeth.1923
macs2 2.1.2 https://doi.org/10.1186/gb-2008-9-9-r137
fastqc 0.11.5 https://www.bioinformatics.babraham.ac.uk/projects/fastqc/
cutadapt 2.1 http://dx.doi.org/10.14806/ej.17.1.200
picard 2.18.11 http://broadinstitute.github.io/picard/
nextflow DSL1 https://doi.org/10.1038/nbt.3820

parameters for read mapping : bowtie default parameters

Flow Cytometry

Plots

Confirm that:

- [x] The axis labels state the marker and fluorochrome used (e.g. CD4-FITC).
- [x] The axis scales are clearly visible. Include numbers along axes only for bottom left plot of group (a 'group' is an analysis of identical markers).
- [ ] All plots are contour plots with outliers or pseudocolor plots.
- [x] A numerical value for number of cells or percentage (with statistics) is provided.

Methodology

Sample preparation: Asynchronous or elutriated DT40 cells were fixed in 75% ethanol and stored at -20°C. On the day of analysis, fixed cells were resuspended at a final concentration of 2.5 x 10^6 cells/mL in 0.1% IGEPAL in PBS (Sigma, #CA-630), 50 µg/ml propidium iodide and 0.5 mg/ml RNase A, and incubated for 30 minutes at room temperature.

Instrument: For cell cycle analysis, we used with a CyanADP cell analyzer (Beckman coulter). For replication timing analysis cells were sorted with an INFLUX 500 cell sorter (Cytopeia, BD Biosciences) or a FACSAria Fusion (BD Biosciences).

Software: Cell cycle analysis was performed using CyAn™ ADP with Summit™ Software and the cell sorting was performed with either the Summit™ Software or the BD FACSDiva™ Software.

Cell population abundance: For cell cycle analysis a total of 20,000 cells were analyzed per sample. For replication timing analysis four S-phase fractions of 50,000 cells are collected from early to late s-phase.

Gating strategy: The gating strategy removes debris and dead cells and cells aggregated in doublets or more

- [x] Tick this box to confirm that a figure exemplifying the gating strategy is provided in the Supplementary Information.
